# Leaf transformation for efficient random integration and targeted genome modification in maize and sorghum

**DOI:** 10.1038/s41477-022-01338-0

**Published:** 2023-02-09

**Authors:** Ning Wang, Larisa Ryan, Nagesh Sardesai, Emily Wu, Brian Lenderts, Keith Lowe, Ping Che, Ajith Anand, Andrew Worden, Daleen van Dyk, Pierluigi Barone, Sergei Svitashev, Todd Jones, William Gordon-Kamm

**Affiliations:** 1 Corteva Agriscience, Johnston, IA USA; 2 Present Address: MyFloraDNA, Woodland, CA USA

**Keywords:** Molecular engineering in plants, Genetic engineering

## Abstract

Transformation in grass species has traditionally relied on immature embryos and has therefore been limited to a few major Poaceae crops. Other transformation explants, including leaf tissue, have been explored but with low success rates, which is one of the major factors hindering the broad application of genome editing for crop improvement. Recently, leaf transformation using morphogenic genes *Wuschel2* (*Wus2*) and *Babyboom* (*Bbm*) has been successfully used for Cas9-mediated mutagenesis, but complex genome editing applications, requiring large numbers of regenerated plants to be screened, remain elusive. Here we demonstrate that enhanced *Wus2*/*Bbm* expression substantially improves leaf transformation in maize and sorghum, allowing the recovery of plants with Cas9-mediated gene dropouts and targeted gene insertion. Moreover, using a maize-optimized *Wus2*/*Bbm* construct, embryogenic callus and regenerated plantlets were successfully produced in eight species spanning four grass subfamilies, suggesting that this may lead to a universal family-wide method for transformation and genome editing across the Poaceae.

## Abstract (web summary)

Enhanced *Wus2*/*Bbm* expression substantially improves leaf transformation, Cas9-mediated gene dropouts and targeted gene insertion in maize, sorghum and other Poaceae species.

## Main

The genetic transformation and regeneration of plants with new phenotypes was a breakthrough in early plant biotechnology and generated high expectations among plant scientists. However, soon after the first report of successful *Nicotiana tabacum* transformation using *Agrobacterium* infection^1^, it became apparent that tobacco is the exception and harnessing the potential of this technology in major crops would be prevented by poor transformation and/or regeneration^2^. Stepwise improvements in transformation have continued for various plant species, but for many of them this process has been limited by recalcitrance to in vitro manipulation. Finding the best combination of transformation method and culture system for each crop has required the evaluation of different explants ranging from single cells such as protoplasts; to embryonic tissues such as meristem, scutellum or cotyledons; to seedling-derived tissues such as apical/axillary meristems, hypocotyl or leaves; and finally to *in planta* alternatives.

Recent development of genome editing technologies based on CRISPR (clustered regularly interspaced short palindromic repeats)–Cas (CRISPR-associated) nucleases further exacerbates the need for improved and efficient transformation processes^3^. RNA-guided Cas nucleases generate targeted double-stranded breaks in the genome, which are repaired either through non-homologous end joining (NHEJ) or homology-directed repair (HDR) pathways. NHEJ is prone to imperfect repair and often results in small insertions or deletions, while HDR can be used to introduce predefined modifications by providing a donor DNA template with regions of homology to the double-stranded break site.

Since the first reports of Cas9-mediated genome editing in tobacco, *Arabidopsis* and rice, the list of edited plant species has continued to expand^4–7^. However, while NHEJ-mediated mutagenesis and gene knockouts are now common practice, other types of genome edits (such as HDR-mediated gene insertions, large-scale deletions, inversions and translocations) occur with much lower frequencies, requiring a high number of plants to be regenerated and screened to find one with the desired modification. This limitation is true for most species in the Poaceae (for example, indica rice, maize, wheat, barley, sorghum and pearl millet), where transformation methods rely on immature embryos and in general remain very species- and/or genotype-dependent, limiting their large-scale deployment. In addition, a consistent year-round supply of immature embryos as a transformation explant requires greenhouse infrastructure that makes it resource- and cost-prohibitive for most academic institutions. Consequently, transformation service facilities have become a necessity^8^. Improved transformation processes with high frequencies of regeneration therefore become a prerequisite for these complex genome editing applications^9^.

A major breakthrough in immature-embryo-based transformation came with the use of the morphogenic genes *Wuschel2* (*Wus2*) and *Babyboom* (*Bbm*), allowing plant regeneration at high frequencies from recalcitrant maize genotypes^10,11^. The use of *Wus2*/*Bbm* also improved transformation in three sorghum varieties, Tx430, Tx623 and P898012 (refs. ^12,13^), and two public maize lines, FFMM^14^ and B104 (ref.^15^). In addition, the expression of *Wus2* alone has been shown to be effective for improving transformation in maize^16^ and genome editing in sorghum^17^. Following these reports, other genes stimulating cell division and the formation of embryogenic tissue have been identified and used with similar outcomes. For example, *Grf4*/*Gif1* (ref. ^18^) and *Wox5* (ref.^19^) have improved immature embryo transformation and regeneration in a wide range of wheat cultivars. However, for some grass species (such as bamboos, *Setaria*, teff and the small millets), seed and immature embryos are simply too small to efficiently isolate, while other crops (such as sugarcane and banana) are vegetatively propagated.

Various research groups have explored alternative starting explants for the transformation of grass species. Except for japonica rice, readily transformed via *Agrobacterium* infection of mature seeds^20^, these groups have used either mature seed-derived embryos^21,22^ or seedling-derived leaf bases^23–26^ to first establish callus cultures, which are then used as the target for *Agrobacterium* infection. The use of *Wus2* and *Bbm* expanded the list of alternative explants for transformation, as demonstrated by the regeneration of fertile transgenic plants using mature embryo slices and leaf-base tissue segments in maize^10^. Using constructs developed by Lowe et al.^10^, recent reports have shown leaf-base transformation in switchgrass^27^ and successful gene mutagenesis in *Eragrostis tef*^28^. However, further improvements are needed to increase transformation frequency, to expand the method within the grass family and to eventually enable more complex genome editing applications.

Here we describe a new combination of promoters regulating the expression of *Wus2* and *Bbm* that stimulate direct rapid formation of somatic embryos and regeneration of T_0_ plants after *Agrobacterium* infection of maize and sorghum seedling-derived early leaf tissue. We also demonstrate that this improved leaf transformation method enables Cas9-mediated genome modifications, including gene dropout and targeted gene insertion in maize. Finally, using a vector design, media and leaf transformation protocol optimized for maize, we demonstrate leaf transformation in a variety of grass species, including rice (both indica and japonica), teff, switchgrass, pearl millet, foxtail millet, barley and rye.

## Results

Our previous work using *Wus2*/*Bbm* established both the starting point and our new objective for the optimization of leaf transformation. Lowe et al.^10^ determined that the use of *Wus2* regulated by the *Nopaline synthase* (*Nos*) promoter and *Bbm* regulated by *Ubiquitin* (*Ubi*) produced embryogenic callus that after 10–12 weeks could produce transgenic plants for recalcitrant maize genotypes (our starting point). Later, Lowe et al.^11^ demonstrated that by providing a morphogenic pulse using a different set of promoters regulating *Wus2* and *Bbm* expression (*Axig1* and *Pltp*, respectively), immature embryos would rapidly (in 7–14 days) produce somatic embryos that could be regenerated into T_0_ plants. Our new objective for leaf transformation was therefore the identification and evaluation of promoters and tissue culture conditions that allow rapid leaf transformation and plant regeneration with efficiencies that allow complex genome editing applications, such as targeted gene insertion. The genetic components used in expression cassettes within the transfer DNA (T-DNA) in different plasmids are listed in Supplementary Table 1.

### Identification of promoters that stimulate rapid somatic embryo formation in leaf tissue

**Fig. 1:**
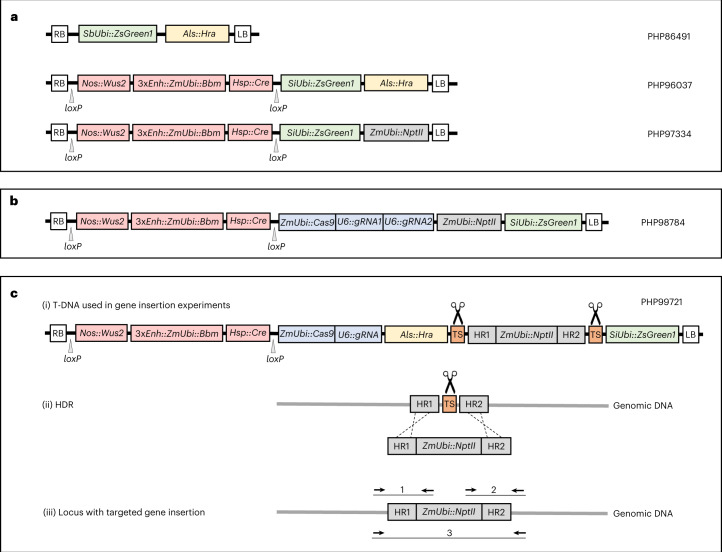
Schematic depiction of T-DNA constructs used in *Agrobacterium*-mediated maize leaf transformation experiments. **a**, T-DNA vectors used in random integration experiments: PHP86491 is a control vector containing only visible (Zs*Green1*) and selectable (*Hra*) marker genes; PHP96037 and PHP97334 are vectors containing morphogenic genes (*Wus2* and *Bbm*), *Cre* recombinase, a visible marker gene (Zs*Green1*) and either *Hra* or *NptII* as a selectable marker gene, respectively. **b**, T-DNA vector used for *Cas9*-mediated *Wx1* dropout containing morphogenic genes (*Wus2* and *Bbm*), *Cre* recombinase, *Cas9* and two gRNA expression cassettes, visible (Zs*Green1*) and selectable (*NptII*) marker genes. **c**, Depiction of *Cas9*-mediated targeted gene insertion experiment, including (i) the T-DNA vector containing the morphogenic genes (*Wus2* and *Bbm*), *Cre* recombinase, *Cas9* and gRNA expression cassettes, a selectable marker gene (*Hra*), the donor cassette comprising the second selectable marker gene (*NptII*), homology arms (HR1 and HR2) flanked by Cas9 cut sites (or target sites (TS)) and a visible marker gene (Zs*Green1*); (ii) targeted gene insertion via HDR upon Cas9 cleavage of the genomic target site and both target sites within the T-DNA to release the donor sequence and (iii) the targeted gene insertion locus with the positions of PCR primer pairs used for detecting the HR1 (1) and HR2 (2) junctions, and the long-PCR product across the entire insertion (3). RB and LB = the right and left border sequences of the *Agrobacterium* T-DNA.

**Fig. 2:**
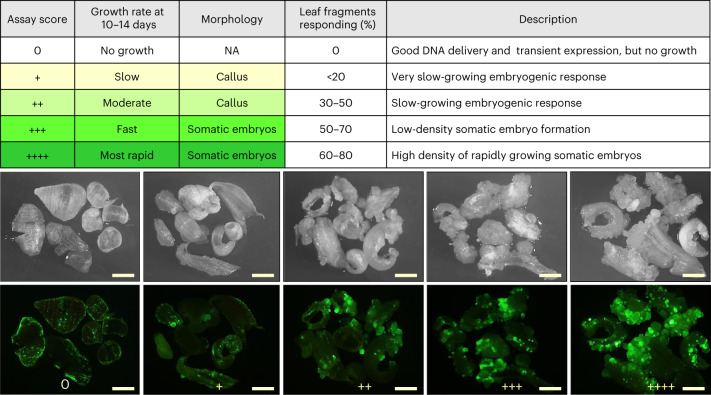
Scoring method for evaluating somatic embryo formation in transformed leaf tissue. The images show examples of scoring the growth of fluorescent cells and somatic embryos 14 days after *Agrobacterium* infection. The images are representative of three independent experiments. Scale bar = 1 mm. NA = not applicable since no resulting tissue grew.

To evaluate promoter combinations with the objective of improving and accelerating the embryogenic response in leaves, we used *Agrobacterium tumefaciens* strain LBA4404 TD THY- harbouring helper plasmid PHP71539 (pVIR9; ref. ^29^) and a binary donor vector (see PHP96037 as a general example; Fig. 1a) to transform leaf tissue from the lower portion of seedlings of Pioneer maize line PH1V69. The amount of callus or direct somatic embryos produced from leaf fragments was used as the criterion to evaluate various combinations of promoters regulating the expression of *Wus2* and *Bbm* (the scoring is described in Fig. 2). The results of these experiments are summarized for maize inbred PH1V69 in Table 1. In general, weaker expression of *Wus2*/*Bbm* resulted in no embryogenesis in the leaf tissue, while different combinations of stronger and/or constitutive promoters produced an increasing embryogenesis response.

**Table 1:** Evaluation of the leaf transformation response for inbred PH1V69, scored 14 days after *Agrobacterium* infections

Plasmid	Prom./term. for *Wus2*	Prom./term. for *Bbm*	*Cre* (upstream or downstream)	Selectable marker	SE TXN response^a^
PHP83652	*Axig1*/*In2*	*Pltp*/*T28*	*Hsp17* (down)	*Hra*	0
PHP83475	*Pltp*/*In2*	(No *Bbm*)	*Hsp17* (down)	*Hra*	0
PHP104052	(No *Wus2*)	3x*Enh–Ubi*/*T28*	*Hsp17* (down)	*NptII*	0
PHP83621	*Pltp*/*In2*	*Pltp*/*T28*	*Glb1* (down)	*Hra*	0
PHP81856	*Axig1*/*In2*	*Pltp*/*T28*	*Rab17*^e^ (down)	*NptII*	0
PHP96730	*Sweet11*/PII	*Ubi*/*PinII*	*Hsp17* (down)	*Hra*	0
PHP96731	*Diurnal10*/*PinII*	*Ubi*/*PinII*	*Hsp17* (down)	*Hra*	0
PHP81855	8x*DR5–35S*/*PinII*	*Pltp/T28*	*Rab17* (up)	*NptII*	+
PHP94636	8x*DR5–35S*/*PinII*	*Pltp*/*T28*	*Rab17* (down)	*NptII*	+
PHP93559	*Nos*/*In2*	*Ubi*/*Ubi*	*Hsp17* (up)	Pmi	+
PHP93926	*Nos*/*In2*	*Ubi*/*T28*	*Hsp17* (up)	*Hra*	+
PHP94684	*Ubi*/*In2*	*Ubi*/*Ubi*	*Hsp17* (up)	*Hra*	+
PHP94685	3x*Enh–Ubi*/*In2*	3x*Enh–Ubi*/*Ubi*	*Hsp17* (up)	*Hra*	+
PHP95073	*Pepc1*/*PinII*	*Ubi*/*PinII*	*Hsp17* (down)	*Hra*	+
PHP95205	*Rubisco Ssu*/*PinII*	*Ubi*/*PinII*	*Hsp17* (down)	*Hra*	+
PHP95394	*Cab*/*PinII*	*Ubi*/*PinII*	*Hsp17* (down)	*Hra*	+
PHP95074	*Diurnal12*/*PinII*	*Ubi*/*PinII*	*Hsp17* (down)	*Hra*	+
PHP96032	*Diurnal11*/*PinII*	*Ubi*/*PinII*	*Hsp17* (down)	*Hra*	+
PHP95393	*CSVMV*/*PinII*	*Ubi*/*PinII*	*Hsp17* (down)	*Hra*	+
PHP95987	*SCBV*/*PinII*	*Ubi*/*PinII*	*Hsp17* (down)	*Hra*	+
PHP81857	*Nos*/*In2*	*Pltp*/*T28*	*Rab17* (down)	*NptII*	++
PHP98564	*Pltp*/*In2*	*Ubi*/*PinII*	*Hsp17* (down)	*Hra*	++
PHP98565	*Pltp*/*In2*	3x*Enh–Ubi*/*T28*	*Hsp17* (down)	*Hra*	++
PHP93739	*Nos*/*In2*	*Ubi*/*Ubi*	*Hsp17* (up)	*Hra*	++
PHP93738	*Actin*/*In2*	*Ubi*/*T28*	*Hsp17* (up)	*Hra*	++
PHP94715	8x*DR5–35S*/*PinII*	*Ubi*/*PinII*	*Rab17* (down)	*NptII*	++
PHP97335	*Nos*/*In2*	3x*Enh–Ubi*/*T28*	*Hsp17* (down)	*NptII* (up)	++
PHP81858	*Nos*/*PinII*	*Ubi*/*PinII*	*Rab17* (down)	*NptII*	++
PHP97978	*Nos*/*In2*	*Ubi*/*T28*	*Hsp17* (down)	*NptII*	++
PHP95385	*Actin*/*In2*	*Ubi*/*T28*	*Hsp17* (down)	*Hra*	+++
PHP96031	*Gos2*/*PinII*	*Ubi*/*PinII*	*Hsp17* (down)	*Hra*	+++
PHP97417	*Ubi*/*In2*	3x*Enh–Ubi*/*T28*	*Hsp17* (down)	*Hra*	+++
PHP93925	*Ubi*/*In2*	3x*Enh–Ubi*/*Ubi*	No *Cre*	*Hra*	++++
PHP96277	*Actin*/*In2*	3x*Enh–Ubi*/*T28*	*Hsp17* (down)	*Hra*	++++
PHP93933	*Nos*/*In2*	3x*Enh–Ubi*/*T28*	No *Cre*	*Hra*	++++
PHP96037	*Nos*/*In2*	3x*Enh–Ubi*/*T28*	*Hsp17* (down)	*Hra*	++++
PHP97334	*Nos*/*In2*	3x*Enh–Ubi*/*T28*	*Hsp17* (down)	*NptII* (down)	++++
PHP99784^b^	*Nos*/*In2*	3x*Enh–Ubi*/*T28*	*Hsp17* (down)	*NptII* (down)	++++
PHP99721^c^	*Nos*/*In2*	3x*Enh–Ubi*/*T28*	*Hsp17* (down)	*NptII* (down)	++++
PHP103735^d^	*Nos*/*In2*	3x*Enh–Ubi*/*T28*	*Hsp17* (down)	*NptII* (down)	++++

Plasmid T-DNA configurations used different combinations of promoters and terminators (prom./term.) driving *Wus2* or *Bbm* expression, different promoters for *Cre* expression, three different selectable markers and altered spatial order of expression cassettes within the T-DNA.

^a^The SE TXN response is the relative stimulation of green-fluorescent somatic embryos (SEs) observed 14 days after *Agrobacterium* infection, measured as the proportion of leaf fragments that produce SEs and the abundance of SEs on each leaf piece.

^b^PHP99784 contained the same *loxP*/*Wus2*/*Bbm*/*Cre*/*loxP* as found in PHP97334, with the addition of *Cas9* and gRNA.

^c^PHP99721 contained the same *loxP*/*Wus2*/*Bbm*/*Cre*/*loxP* as found in PHP97334, with the addition of *Cas9*, gRNA and donor template for HDR into inbred PH1V69.

^d^PHP103735 contained the same *loxP*/*Wus2*/*Bbm*/*Cre*/*loxP* as found in PHP97334, with the addition of *Cas9*, gRNA and donor template for HDR into inbred PHR03.

^e^The medium for inducing the expression of *Cre* in *Rab17* constructs is 13266P plus 0.1 mM ABA (Supplementary Table 15c).

It was known from previous work that the combination of the *Axig1* and *Pltp* promoters produces a pulse of *Wus2* and *Bbm* expression, respectively, which was sufficiently strong in the immature embryo scutellar epithelium to stimulate somatic embryos within 7–14 days^11^. However, in leaf tissue this combination (PHP83652) only produced small clusters of fluorescing cells without further growth. Likewise, two constructs using diurnally regulated promoters (*Sweet 11* or *Diurnal 10*) driving *Wus2* expression along with *Ubi*::*Bbm* did not produce a growth response (PHP96730 and PHP96731, respectively). A weak growth response (+) was observed with a variety of promoter combinations, which included an auxin-inducible promoter (8x*DR5* in PHP81855 and PHP94636); photosynthetic promoters such as *PepC1*(PHP95073), *Rubisco Ssu* (PHP95205) and *Cab* (PHP95394); diurnal promoters such as *Diurnal 12* (PHP95074) and *Diurnal 11* (PHP96032); or two viral promoters, *Cassava Vein Mosaic Virus* (*CSVMV*) (PHP95393) and *Sugarcane Bacilliform Virus* (*SCBV*) (PHP95987), in general being too transient or too weak to elicit rapid embryogenic growth. A more consistent callus growth response (++) was observed with (1) constitutively expressed *Wus2* with transient *Bbm* expression (PHP81857), (2) transient *Wus2* with constitutive *Bbm* (PHP94715, PHP98564 and PHP98565) or (3) our original *Nos*::*Wus2*/*Ubi*::*Bbm* combination (PHP81858 or PHP97978). Three promoter combinations to drive the respective expression of *Wus2* and *Bbm* (*Actin*/*Ubi* in PHP95385, *Gos2*/*Ubi* in PHP96031 or *Ubi*/3x*Enh–Ubi* in PHP97417; 3xEnh, three consecutive viral enhancers from *Figwart Mosaic Virus* (FMV), *Peanut Chlorotic Streak Virus* (PCSV) and *Mirabilis Mosaic Virus* (MMV); Supplementary Table 1) resulted in more rapid embryogenic growth with some somatic embryo formation within 14 days. Finally, various constitutive promoters for *Wus2* (*Ubi*, *Actin* and *Nos*) along with 3x*Enh–Ubi*::*Bbm* (PHP93925 to PHP103735) (Table 1) produced rapid somatic embryo formation in 60–80% of leaf fragments. The presence of 3x*Enh–Ubi* promoter driving *Bbm* expression (PHP97334 and PHP96277) significantly increased transcript levels not only of *Bbm* but also of *Wus2* compared with PHP97978 and PHP95385 with *Ubi*::*Bbm* (Supplementary Table 2). We also observed that having any expression cassette upstream of *Wus2* reduced the growth response, as noted in two specific examples. First, PHP97335 with *Ub*i::*NptII* (neomycin phosphotransferase II) upstream of *Nos*::*Wus2* produced a moderate growth response, while PHP97334 (where *Ubi*::NptII is downstream) generated a strong rapid somatic embryo response; second, PHP93738 with a *Heat Shock Protein17* promoter (*Hsp17*) driving *Cre* upstream of *Actin*::*Wus2* resulted in a moderate callus response, while PHP95385 (where *Hsp17*::*Cre* is downstream) produced a rapid somatic embryo response. Of all constructs tested, PHP96037 and PHP97334, both with *Nos*::*Wus2* and 3x*Enh–Ubi*::*Bbm* but two different selectable markers, consistently demonstrated a strong and rapid growth response and were chosen for further experiments.

### *Agrobacterium*-mediated transformation of seedling-derived leaf tissue in maize

**Fig. 3:**
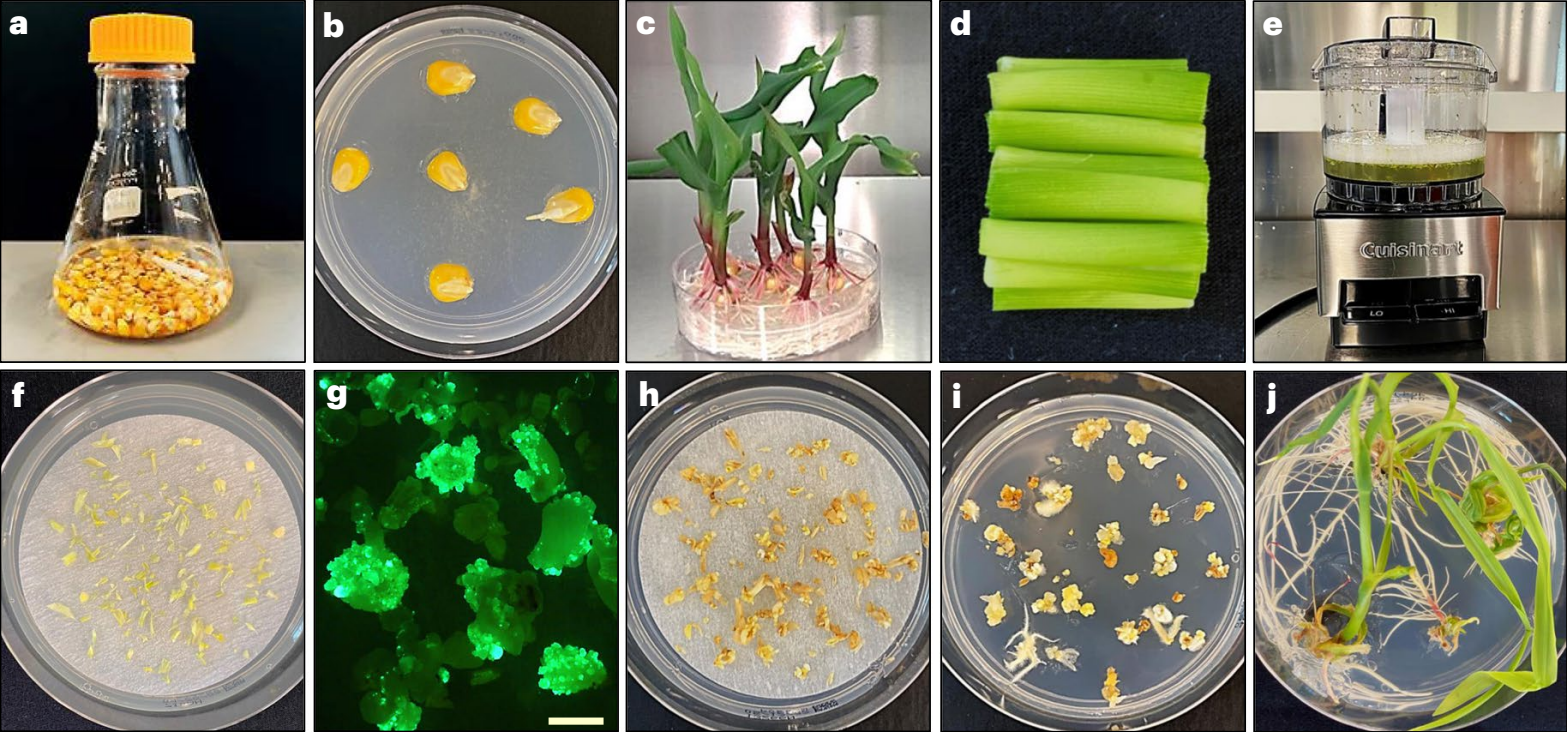
The leaf transformation method used for maize. **a**, Surface sterilization of maize seeds. **b**, Maize seeds on germination medium. **c**, Fourteen-day-old maize seedlings used as the explant source. **d**, Preparation of 3 cm segments of the leaf whorl directly above the mesocotyl followed by slicing the tissue longitudinally (not shown). **e**, The tissue was placed in 100 ml of *Agrobacterium* suspension in the mini-Cuisinart blender and subjected to ten brief pulses to further slice up the leaf tissue. After a 20-minute incubation in the *Agrobacterium* suspension, the liquid was poured through a metal sieve to collect the leaf fragments, which were briefly blotted onto dry filter paper to wick away excess *Agrobacterium* (not shown). **f**, Leaf fragments distributed onto filter paper on the solid RM. **g**, Fluorescent somatic embryo growth observed 7–10 days after *Agrobacterium* infection (the image is representative of over 100 independent experiments; scale bar, 1 mm). **h**, After culturing on the RM, leaf tissue was transferred to selection medium. **i**, Following three weeks on filter papers on selection medium, the tissue was subjected to 45 °C/70% RH for two hours (not shown), transferred to maturation medium and cultured for an additional three weeks. **j**, Formation of T_0_ regenerants after two weeks on rooting medium.

To evaluate the overall leaf tissue transformation process (including T_0_ plant regeneration), the binary vectors containing *Wus2*, *Bbm*, *Cre*, a fluorescent marker gene (Zs*Green1*) and a selectable marker gene (*Highly-Resistant* Zm*Als* (*Hra*) (PHP96037) or *NptII* (PHP97334)) were compared with a control vector (PHP86491) lacking *Wus2*/*Bbm* (Fig. 1a). *LoxP* sites were introduced, allowing the excision of *Wus2/Bbm* and *Cre* expression cassettes prior to T_0_ plant regeneration. Seedling preparation and the transformation process are shown in Fig. 3. Surface-sterilized seed was germinated on agar-solidified medium for 14 days (Fig. 3a–c), and then approximately 3 cm of leaf tissue above the mesocotyl (Fig. 3d) was bisected longitudinally and mechanically chopped in a food processor while immersed in the *Agrobacterium* suspension (Fig. 3e). The leaf fragments were distributed on filter papers and placed on co-cultivation medium for 24 hours and then resting medium (RM) for 7 days (Fig. 3f). Green-fluorescent somatic embryo formation was observed from the leaf fragments approximately 7–10 days after infection (Fig. 3g). After three weeks of selection on the filter papers (Fig. 3h), the plates containing the tissue were moved into a 45 °C incubator for two hours to induce Cre-mediated excision, and then individual pieces of healthy embryogenic tissue were transferred onto maturation medium (Fig. 3i) and finally onto rooting medium (Fig. 3j), where plantlet development was completed before transfer to soil in the greenhouse.

**Extended Data Fig. 1:**
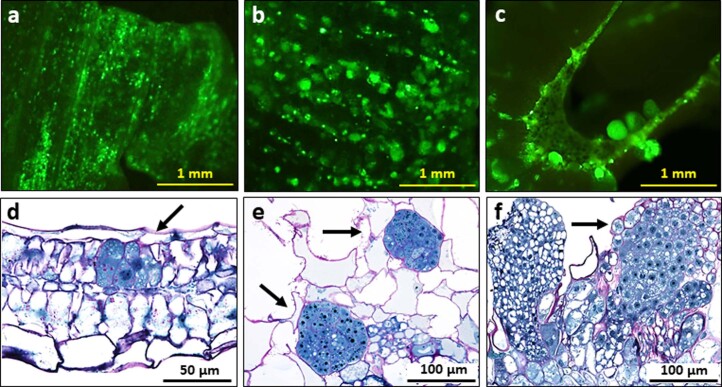
Early growth of somatic embryos. **a**, Fluorescence image of transient ZsGreen1 expression four days after *Agrobacterium* infection with PHP97334. **b**, Growing, independent fluorescent foci as somatic embryos begin growing nine days post-infection. **c**, Higher magnification at nine days post-infection showing fluorescent somatic embryos emerging from the surface of the leaf. **d-f**, Micrographs of transverse sections of leaf tissue stained with PAS and counter-stained with aniline blue black, showing **d**, small clusters of dividing cells depicting early developing somatic embryos (arrows) in leaf segments five days after infection, **e**, cluster of dividing cells ready to emerge through the epidermal layer in leaf segments ten days after infection, and **f**, larger somatic embryos pushing through the surface of the leaf (arrows) in leaf segments 15 days after infection. See end of Supplementary section for histology methods. Images are representative of over ten independent experiments.

The origin of somatic embryos within transformed leaf pieces (Extended Data Fig. 1a,b) was inferred on the basis of histological observations of the tissue, which clearly showed multicellular clusters appearing to initiate from internal leaf cells (Extended Data Fig. 1d–f). These clusters continued to grow as somatic embryos finally pushed out through the surface of the leaf (Extended Data Fig. 1c,f).

The results of leaf tissue transformation for maize stiff-stalk (SS) inbred PH1V69 from four experiments using vector PHP96037 are shown in Table 2a. In four replicates, each starting with ten seedlings used to generate the leaf fragments, the number of transgenic T_0_ plants recovered ranged from six (Experiments 1 and 4) to 14 (Experiment 2), resulting in a mean transformation frequency of 98 ± 43% (calculated on the basis of the number of T_0_ regenerants per starting seedling). In three replicate treatments with the control vector PHP86491 (Fig. 1a), T-DNA delivery comparable to other *Ubi*::Zs*Green1*-containing plasmids was observed, but no green-fluorescent somatic embryo formation occurred in these leaf pieces, and no transgenic T_0_ plants were recovered using the same transformation protocol.

**Table 2:** Transformation frequency (percentage of transgenic T_0_ plants per number of starting seedlings), single-copy frequency and escape frequency after *Agrobacterium* infection of maize inbred PH1V69 leaf segments (a–c) or sorghum variety Tx430 (d)

Treatment	Number of seedlings	Number of T_0_ plants	T_0_ freq. (%)^a^	Number of single-copy T-DNA	SC/BB-free freq. (%)^b^	Overall SC/BB-free freq. (%)^c^	Number of escapes	Escape freq. (%)^d^
**(a) Maize inbred PH1V69 using PHP96037 (Hra with ethametsulfuron selection)**								
Exp. 1	10	6	60	0	0	0	0	0
Exp. 2	10	14	140	8	57	80	1	7
Exp. 3	10	13	130	5	38	50	0	0
Exp. 4	10	6	60	3	50	30	2	33
**Mean (s.d.)**			**98 (43)**		**36 (25)**	**40 (29)**		**10 (16)**
**(b) Inbred PH1V69 seedlings with different ancymidol pretreatments (PHP97334 with G418 selection)**								
0 mg l^−1^^e^	33	36	109	19	53	58	1	3
0 mg l^−1^	32	31	97	19	61	59	1	3
0 mg l^−1^	0	NA	NA	NA	NA	NA	NA	NA
**Mean (s.d.)**			**103 (6)**		**57 (4)**	**58 (0.5)**		**3**
2 mg l^−1^^e^	33	118	358	51	43	155	5	4
2 mg l^−1^	32	97	303	67	69	209	3	3
2 mg l^−1^	28	66	236	27	41	96	7	11
**Mean (s.d.)**			**299 (50)**		**51 (13)**	**153 (46)**		**6 (3.5)**
4 mg l^−1^^e^	33	83	252	45	54	136	4	5
4 mg l^−1^	32	81	253	51	63	159	5	6
4 mg l^−1^	32	75	234	51	68	159	3	4
**Mean (s.d.)**			**246 (9)**		**62 (6)**	**151 (11)**		**5 (0.8)**
**(c) Inbred PH1V69 seedlings, with either 2** **mg** **l**^**−1**^ **ancymidol (ANC) or ANC** + **3** **h heat treatment (ANC** + **HT) (PHP99721 with** ***NptII*** **and G418 selection)**								
ANC^f^	25	61	244	26	43	104	7	11
ANC	25	48	192	16	33	64	3	6
ANC	25	111	444	54	49	216	8	7
ANC	23	34	148	14	41	61	4	12
ANC	25	68	272	24	35	96	3	4
**Mean (s.d.)**			**260 (101)**		**40 (6)**	**108 (56)**		**8 (3)**
ANC + HT^g^	25	138	552	59	43	236	13	9
ANC + HT	23	112	487	54	48	235	7	6
ANC + HT	22	159	723	77	48	350	9	6
ANC + HT	25	134	536	61	46	244	12	9
ANC + HT	18	90	500	43	48	239	2	2
**Mean (s.d.)**			**560 (85)**		**46 (2)**	**261 (45)**		**6.4 (2.6)**
**(d) Sorghum variety Tx430 using PHP96037 (Hra with imazapyr selection)**								
RM^h^	13	18	138	7	39	54	2	11
RM	15	46	307	19	41	127	5	11
RM	15	14	93	5	36	33	0	0
RM	16	20	125	6	30	38	3	15
**Mean (s.d.)**			**166 (96)**		**37 (5)**	**63 (38)**		**9 (6)**
RM + CB^i^	15	65	433	26	40	173	2	3
RM + CB	15	56	373	18	32	120	2	4
RM + CB	15	32	213	7	22	47	0	0
RM + CB	16	27	169	13	48	81	9	33
**Mean (s.d.)**			**297 (126)**		**35 (11)**	**105 (47)**		**10 (13)**

^a^Calculated on the basis of the number of putative T_0_ plants regenerated relative to the number of starting seedlings used for transformation.

^b^SC/BB-free, single-copy backbone-free. Calculated on the basis of the number of T_0_ plants containing a single copy of the T-DNA with no expression plasmid backbone (BB), relative to the number of T_0_ plants analysed.

^c^Overall SC/BB-free frequency is based on the number of T_0_ plants containing a single copy of the T-DNA with no expression plasmid backbone, relative to the number of starting seedlings transformed.

^d^Calculated on the basis of the number of non-transgenic plants relative to the total number of T_0_ plants analysed.

^e^Concentration of ancymidol added to medium during the 14-day seedling germination period.

^f^Two mg l^−1^ ancymidol added to medium during the 14-day seedling germination period.

^g^Two mg l^−1^ ancymidol added during 14-day germination and seedling growth, with a three-hour exposure to 45 °C/70% RH before *Agrobacterium* infection.

^h^Resting medium.

^i^Resting medium plus CuSO4 and benzylaminopurine (BAP). NA = not applicable since no third replicate was performed for this treatment.

These leaf transformation experiments produced a high percentage (from 0% to 57% with a mean of 36 ± 25%) of high-quality T_0_ regenerants with a single copy of the integrated T-DNA (containing selectable and fluorescent marker genes) with no backbone sequences inserted elsewhere, as determined by quantitative PCR (qPCR). Comparing this number of high-quality transgenic events with the number of starting seedlings used in these experiments provided a clear measure of overall efficiency for producing single-copy T_0_ plants, with a mean frequency of 40 ± 29%.

Single-copy transgenic regenerants were grown to maturity in the greenhouse. The T_0_ plant phenotype was comparable to that of wild-type (non-transgenic) seed-derived plants. T_0_ plants were then either self-pollinated or crossed with wild-type plants and produced on average 172 (11 ears) and 195 (17 ears) seeds per ear, respectively (Supplementary Table 3). Thirty-six single-copy T_0_ plants were also analysed using Southern-by-Sequencing (SbS) technology^30^ (Supplementary Table 4). Twenty-seven plants (75%) were ‘SbS-pass’, indicating that a single intact copy of the integrated T-DNA with *Wus2*, *Bbm* and *Cre* excised was present, with no unlinked, independently segregating small DNA fragments detected. The remaining 25% failed due to an assortment of issues including single nucleotide polymorphisms, linked or unlinked fragments, plasmid backbone or mismatched border sequences (Supplementary Table 4).

### Ancymidol during maize seedling growth and heat treatment improves leaf transformation frequency

During rapid seedling growth, leaves often contacted the lid of the growth container, resulting in tissue necrosis and phenolic production. To mitigate this problem, we added ancymidol, an antagonist of gibberellin-regulated elongation^31^, to the germination and seedling growth medium to produce shorter, more compact seedlings. In addition, we observed that ancymidol pretreatment of seedlings improved transformation frequency as demonstrated using PHP97334 vector, which contained a T-DNA with the same *Wus2*, *Bbm*, *Cre* and Zs*Green1* expression cassettes as in PHP96037, except for an *SiUbi*::*NptII* replacing the SbAls::Zm*Hra* as a selectable marker gene (Fig. 1a).

For three replicates of this experiment performed using three separate plantings of seedlings on three different media (Table 2b), seedlings grown in the absence of ancymidol (control) produced a mean transformation frequency of 103 ± 6%, while seedlings grown on either 2 mg l^−1^ or 4 mg l^−1^ ancymidol resulted in mean transformation frequencies of 299 ± 50% and 246 ± 9%, respectively. The two ancymidol treatments did not significantly differ from each other (*P* = 0.32) but were substantially higher than the control (*P* < 0.02). All three treatments (with 0, 2 and 4 mg l^−1^ ancymidol pretreatments) produced T_0_ plants in which a similar proportion contained a single copy of the integrated T-DNA.

As previously observed for immature embryos^32,33^, temperature treatment could positively impact downstream transformation results. On the basis of this idea, a subset of PH1V69 seedlings grown on media supplemented with 2 mg l^−1^ ancymidol at 28 °C were incubated at 45 °C and 70% relative humidity (RH) for three hours, followed by leaf tissue collection and mechanical fragmentation in the presence of *Agrobacterium* suspension. As shown in Table 2c, the control treatment resulted in a mean transformation frequency of 260 ± 101%. Pretreating seedlings at 45 °C for three hours resulted in a significantly higher transformation frequency of 560 ± 85% (*P* = 0.002).

### Public SS inbred line B104 and Pioneer non-SS maize inbreds respond positively to leaf transformation

To assess whether this method works in the commonly used maize inbred B104, seedlings grown on 2 mg l^−1^ ancymidol with the three-hour 45 °C/70% RH pretreatment were used as the tissue source, and transformation was carried out as described for PH1V69. For two replicates starting with seven seedlings for each experiment, the numbers of T_0_ plants produced were 12 and 13 for transformation frequencies of 171% and 186%, respectively (Supplementary Table 5). Of these total 12 and 13 T_0_ plants, 8 and 7 had single-copy T-DNA insertions, respectively, and further analysis showed that four of eight and five of seven events had no extraneous plasmid backbone sequence (Supplementary Table 5).

The leaf transformation method developed for our SS PH1V69 was also tested in four Pioneer non-SS (NSS) maize inbreds using PHP97334 (containing *Nos*::*Wus2* + 3xEnh–*Ubi*::*Bbm*), starting with 21–24 seedlings per genotype with no replicates. Inbreds PH4257 and PNSS01 produced fewer somatic embryos within the first 14 days after *Agrobacterium* infection, resulting in a lower transformation response ranking compared with the SS inbred PH1V69 (Supplementary Table 6). As a result, PH4257 produced no transgenic plants, and only three T_0_ plants were regenerated for PNSS01 (13% relative to the number of starting seedlings).

In contrast, the other two NSS inbreds (1PYWK36 and 1PEEA63) demonstrated strong early somatic embryo responses similar to inbred PH1V69. Nevertheless, for these two inbreds, the conversion of these somatic embryos into T_0_ plants was considerably less efficient, producing only ten and eight T_0_ plants (48% and 38% transformation frequency, respectively). However, when a different construct (PHP96277) containing Actin-regulated *Wus2* was used, the number of regenerated T_0_ plants increased to 20 and 40 (95% and 190%) for inbreds 1PYWK36 and 1PEEA63, respectively. While the results for these four inbreds were not replicated and are thus preliminary, taken together, we demonstrated that the leaf transformation method worked for six of the seven maize inbreds tested.

### Sorghum is amenable to *Agrobacterium*-mediated seedling-derived leaf tissue transformation

To test the maize leaf transformation method in another crop, we used the same *Agrobacterium* strain, constructs, growth of seedlings, preparation of leaf material, infection, co-culture, resting culture, selection, *Wus2*/*Bbm* excision, somatic embryo maturation and rooting media for sorghum leaf transformation. Even though PHP96037 contained the *Hra* gene, no selection was used in this set of experiments because no background regeneration was observed from leaf tissue in initial observations in the absence of *Wus2*/*Bbm*. In addition, in this set of experiments we compared two resting media (RM): 13266P medium plus 50 mg l^−1^ meropenem and RM + CB (100 μM cupric sulfate and 0.5 mg l^−1^ benzylaminopurine).

The results from four sorghum (Tx430 variety) experiments are shown in Table 2d. Sorghum leaf transformation using PHP96037 vector resulted in high regeneration frequencies, calculated on the basis of the number of transgenic T_0_ plants recovered per starting seedling, with a mean frequency of 166 ± 96% and 297 ± 126% for RM and RM + CB, respectively, with no significant difference between the two media (*P* = 0.15). The control treatment with no *Wus2*/*Bbm* in the T-DNA (PHP86491) did not produce regenerated plants. The mean frequency of high-quality (single-copy T-DNA/backbone-free) T_0_ sorghum plants when transformed with PHP96037 was between 35% and 37%, with no significant difference between the two media (*P* = 0.87).

Twenty-three single-copy T_0_ sorghum plants produced through rapid leaf transformation were grown to maturity in the greenhouse. These plants were healthy, displaying an overall phenotype similar to that of wild-type Tx430 plants. SbS analysis demonstrated that of 23 T_0_ plants, 17 contained a single copy of the integrated T-DNA (Supplementary Table 4). Twenty-one T_0_ plants (91%) were fertile, with seed sets ranging from 667 to 3,255 seeds per head and a mean of 2,057 ± 900 seeds per head (Supplementary Table 3).

### Leaf transformation facilitates gene dropout in maize

To assess the efficiency of gene dropout in seedling-derived leaf tissue of maize inbred PH1V69, we used a T-DNA vector (PHP98784, Fig. 1b) that contained the previously described components complemented with Cas9 and two guide RNA (gRNA) expression cassettes targeting two sites flanking the endogenous *Wx1* gene as previously described by Gao et al.^34^. For these experiments, 2 mg l^−1^ ancymidol was used in the germination medium for inbred PH1V69, and a three-hour seedling heat pretreatment was applied prior to *Agrobacterium* infection.

The results of three *Wx1* dropout experiments are summarized in Table 3. High transformation frequencies of 636%, 544% and 363% for a mean of 514 ± 139% were observed. Quantitative PCR analysis performed on T_0_ plants demonstrated dropout frequencies of 4.4%, 8.1% and 8.0% for these three experiments, respectively, for a mean of 6.8 ± 1.7%.

**Table 3:** Transformation efficiency and *Cas9*-mediated *Wx1* gene dropout frequency in maize inbred PH1V69 after heat pretreatment prior to *Agrobacterium*-mediated leaf transformation using PHP98784

Exp. No.	Number of seedlings	Number of T_0_ plants	Transformation frequency (%)^a^	Number of *Wx1* dropouts	Dropout frequency (%)^b^
1	25	159	636	7	4.4
2	25	136	544	11	8.1
3	24	87	363	7	8.0
**Mean (s.d.)**			**514 (139)**		**6.8 (1.7)**

^a^Transformation frequency was calculated as (number of T_0_ plants/number of seedlings) × 100.

^b^Dropout frequency was calculated as (number of *Wx1* dropouts/number of T_0_ plants) × 100.

### HDR-mediated targeted gene insertion is efficiently facilitated in maize using leaf transformation

To test HDR-mediated targeted gene insertion in maize leaf cells, two nearly identical T-DNA vectors were constructed (PHP99721 and PHP103735). Both vectors contained *Wus2*, *Bbm*, *Cre*, *Cas9*, gRNA, a donor template containing a selectable marker gene (*NptII*) flanked with homology arms and sequences identical to the genomic target site (at 53.66 cM on Chr1 (ref. ^35^)), a second selectable marker gene (*Hra*) and Zs*Green1* (PHP99721; Fig. 1c(i)). The two vectors differed in the homology arm sequences specific for the two genotypes used (PHP99721 for PH1V69 and PHP103735 for the Pioneer NSS inbred PHR03). As previously reported^36^, the presence of flanking target sites leads to the release of the donor template from T-DNA upon expression of gRNA and Cas9 (Fig. 1c(ii)). The use of two different selectable marker genes, *NptII* and *Hra*, present inside and outside the donor template, respectively, allowed the comparison of targeted insertion frequencies depending on the selectable marker gene position (Fig. 1c(i)).

For these experiments, seedlings were grown on germination medium with ancymidol. A total of five experiments were conducted using two different Pioneer proprietary genotypes. Experiments 1–4 were performed on inbred PH1V69, while Experiment 5 was conducted using inbred PHR03. In Experiment 1, there was no seedling heat pretreatment, while in Experiments 2 and 3, half of the seedlings were exposed to 45 °C at 70% RH for three hours prior to *Agrobacterium* infection and the other half had no heat pretreatment. In Experiments 4 and 5, all seedlings were subjected to the heat pretreatment. In Experiments 1, 2, 3 and 5, *NptII* (inside the donor template) was used as the selectable marker gene. In Experiment 4, one set of T_0_ plants was regenerated using G418 as the selection agent (for *NptII*), while the second set of plants was regenerated on ethametsulfuron-containing media (for *Hra*).

The results of all five experiments are summarized in Table 4. In Experiments 1–3 when no heat pretreatment was applied, the transformation frequency (the number of T_0_ plants per seedling) was 140%, 262% and 230%, respectively, with a mean of 211 ± 63%. In contrast, in Experiments 2–4 when the temperature pretreatment was used, transformation frequencies increased significantly (*P* = 0.001) to 560%, 570% and 524%, respectively, with a mean of 551 ± 24%. The escape rate (the number of regenerants with no selectable marker gene detected) ranged from 9.0% to 10.9%. T_0_ plants were first analysed for HDR-mediated insertion events by diagnostic HR1- and HR2-junction qPCR (Fig. 1c(iii)) as described in the Methods. T_0_ plants that were qPCR positive for either an HR1 or HR2 junction alone ranged between 5% and 12.6%. T_0_ plants that were positive for both junctions (HR1&HR2 column in Table 4) demonstrated that the targeted insertion frequencies were similar (*P* = 0.71) across the four experiments; 4.5%, 6.8% and 5.2% with no heat pretreatment and 7.0%, 5.6% and 5.0% with pretreatment.

**Table 4:** Analysis of targeted integration via HDR in maize inbred PH1V69 and PHR03 after *Agrobacterium*-mediated transformation with a T-DNA containing *Wus2*/*Bbm*/*Cas9*/gRNA and the Homology Region-flanked (HR1 and HR2) donor template, with the *NptII* selectable marker as part of the donor sequence (T-DNA configuration shown in Fig. 1c)

Experiment number (genotype) Selectable marker (position)	Selective agent	Seedling pretreatment		Number of seedlings	Number of T_0_ plants (TXN freq. %)	Number of escapes (%)	Number of HR1-positive T_0_ plants (%^a^)	Number of HR2-positive T_0_ plants (%^a^)	Number of HR1&HR2-positive T_0_ plants (%^a^)	Number of long-PCR-positive T_0_ plants (%^a^)	Percentage of long-PCR-positive from HR1/HR2-positive T_0_ plants
**Experiment 1 (PH1V69**^**b**^**)**											
NptII (in donor DNA)	G418		None	283	403 (140%)	44 (10.9%)	27 (6.7%)	31 (7.7%)	18 (4.5%)	13 (3.2%)	72%
**Experiment 2 (PH1V69**^**b**^**)**											
NptII (in donor DNA)	G418		None	123	322 (262%)	29 (9.0%)	26 (8.1%)	44 (13.7%)	22 (6.8%)	9 (2.8%)	41%
NptII (in donor DNA)	G418		45 °C, 70% RH 3 h	113	633 (560%)	69 (10.9%)	58 (9.2%)	65 (10.3%)	43 (7.0%)	17 (2.7%)	40%
**Experiment 3 (PH1V69**^**b**^**)**											
NptII (in donor DNA)	G418	None		100	231 (230%)	21 (9.1%)	19 (8.2%)	17 (7.4%)	12 (5.2%)	7 (3.0%)	58%
NptII (in donor DNA)	G418	45 °C, 70% RH 3 h		91	521 (570%)	52 (10.0%)	44 (8.4%)	37 (7.1%)	29 (5.6%)	13 (2.5%)	45%
**Experiment 4 (PH1V69**^**b**^**)**											
NptII (in donor DNA)	G418	45 °C, 70% RH 3 h		72	377 (524%)	37 (9.8%)	27 (7.2%)	30 (8.0%)	19 (5.0%)	10 (2.6%)	53%
Hra (outside donor DNA)	EMS^c^	45 °C, 70% RH 3 h		107	620 (579%)	78 (12.6%)	31 (5.0%)	39 (6.3%)	18 (2.9%)	9 (1.5%)	50%
**Experiment 5 (PHR03**^**d**^**)**											
NptII (in donor DNA)	G418	45 °C, 70% RH 3 h		370	764 (205%)	73 (9.6%)	32 (4.2%)	24 (3.1%)	17 (2.2%)	9 (1.2%)	53%

HR1 and HR2 sequences differed for the two inbreds, with PHP99721 being used for inbred PH1V69 and PHP103735 being used for PHR03.

^a^Percentage of positive T_0_ plants relative to the total number of T_0_ plants analysed; only events with a qPCR cut-off at 0.5 and above are included in the table.

^b^SS genotype.

^c^EMS, ethametsulfuron.

^d^NSS genotype.

Using long PCR that spans the entire insertion site (from the genomic sequence outside the HR1 arm across the integrated donor to the sequence outside the HR2 arm; Fig. 1c(iii)), we were able to detect the frequency of putative perfect insertion events. For both the non-heat-pretreated and the heat-pretreated replicates in Experiments 1–4, the frequencies were very similar: 3.2%, 2.8% and 3.0% and 2.7%, 2.5% and 2.6%, respectively. These results clearly demonstrate that although heat pretreatment resulted in a substantial increase in the initial recovery of T_0_ plants, targeted gene insertion frequencies were very consistent. In addition, the number of long-PCR-positive events relative to the total number of HR1- and HR2-junction-positive T_0_ plants provides an indication of the attrition associated with incomplete and/or complex insertions that occurred through a combination of HDR and NHEJ pathways. These frequencies in Experiments 1–3 were 72%, 41% and 58% for non-heat-pretreated replicates, while for both heat-pretreated replicates the values were 40% and 45%.

Targeted insertion frequencies were also compared using two different selectable marker genes located either inside the donor template (*NptII*), therefore integrating into the target site, or outside the donor template (*Hra*), in which case the selectable marker gene is left in the randomly integrated T-DNA (Experiment 4). Comparing these two treatments, the initial transformation frequency was similar: 524% (with a 9.8% escape frequency) when selected on medium with G418 and 579% (with an escape frequency of 12.6%) when selected on medium with ethametsulfuron. For the treatment with the selectable marker gene inside the donor template, the frequency of HR1&HR2-qPCR-positive events was 5.0%, similar to the frequencies observed in the first three experiments. In comparison, when the selectable marker gene was outside the donor template, the frequency of targeted gene insertion events was lower at 2.9%. The frequencies of long-PCR-positive events for the two groups were 2.6% and 1.5%, respectively.

Experiment 5 was conducted to evaluate targeted gene insertion frequency in the NSS inbred PHR03 (Table 4). Using the same T-DNA design and *NptII* as the selectable marker gene (located inside the donor template), the transformation frequency was 205% (with a 9.6% escape frequency), while the combined HR1&HR2 rate was 2.2% and the final long-PCR frequency was 1.2%.

To evaluate transmission and segregation patterns of the HDR-based targeted integrations, we analysed 32 T_1_ plants of three T_0_ insertion events from Experiment 1 (Table 4) using qPCR for the presence of gene insertion and T-DNA components (*Cas9*, gRNA, *Bbm*, *Wus2*, *Hra* and *NptII*). The results of this analysis are summarized in Supplementary Table 7. All three T_0_ plants showed transmission of the insertion to the T_1_ generation with a segregation ratio of approximately 1:1 (40%, 44% and 56%), consistent with stable Mendelian inheritance of a mono-allelic locus. As expected, approximately 50% of the T_1_ plants with insertion were transgene-free.

T_1_ insertion-positive plants were further analysed by Sanger sequencing to verify the quality and integrity of the insertion. Long PCR products from ten T_1_ selected plants (three or four plants for each of the three T_0_ events) were sequenced and assembled into contigs. Comparison of all ten contigs showed no sequence variation, confirming that every plant had a precise, HDR-mediated gene insertion.

### Leaf transformation across several selected species within the Poaceae

**Fig. 4:**
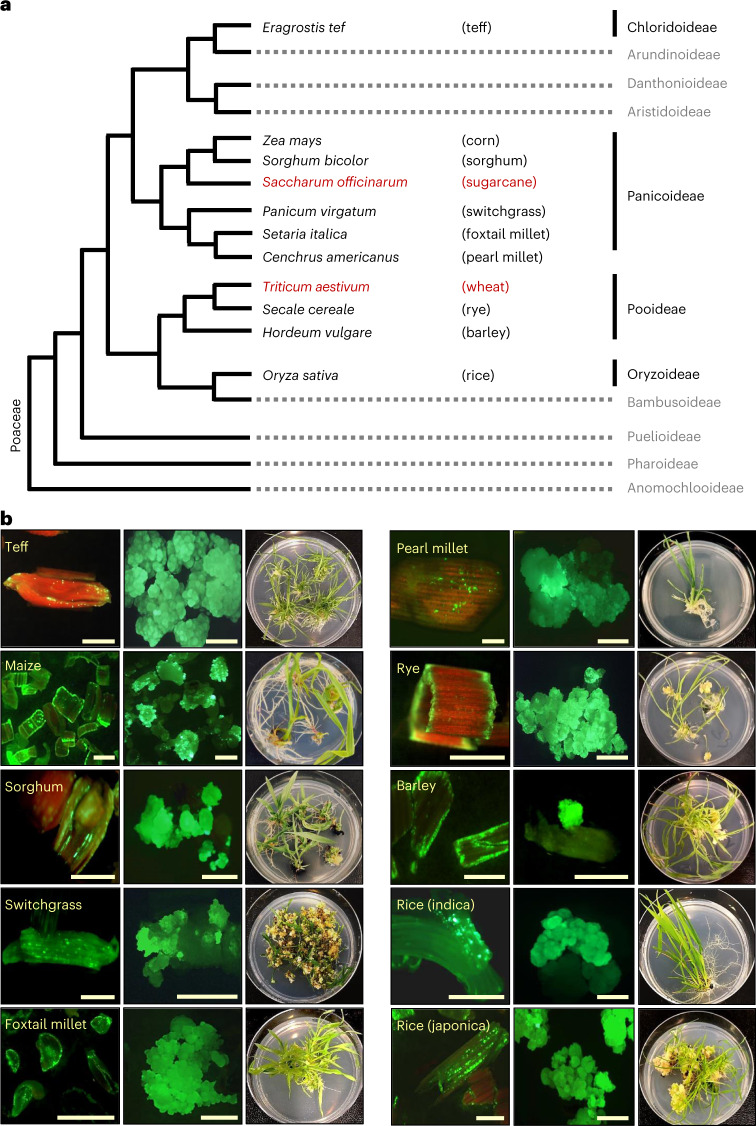
Selected grass species transformed using the maize leaf transformation protocol. **a**, The Poaceae family tree indicating the species used in the experiment and the corresponding subfamilies. Species that were successfully transformed and regenerated, producing T_0_ plants, are shown in black; species that were transformed and produced callus tissue but did not regenerate are shown in red. **b**, Results for all successfully transformed grass species. The images on the left show transient expression of Zs*Green1* three to four days after *Agrobacterium* infection (scale bars, 500 µm), demonstrating the relative T-DNA delivery in various crops; the centre images show examples of green-fluorescent embryogenic callus formation three to four weeks after infection (scale bars, 1 mm); and the images on the right show recovered plantlets with shoots and roots ready for transfer to soil five to eight weeks after infection. The images are representative of three independent experiments.

**Extended Data Fig. 2:**
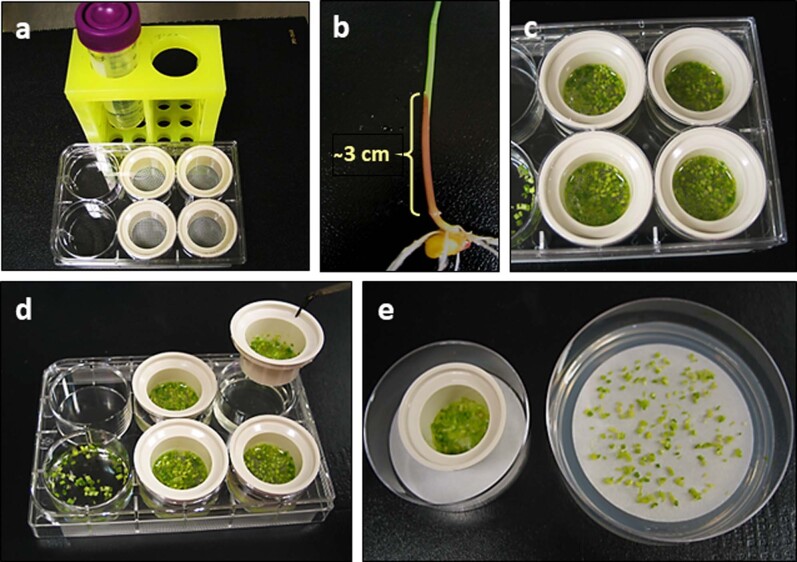
Manual explant preparation and infection. **a**, *Agrobacterium* suspension aliquoted into permeable culture inserts sitting in a six-well culture plate. **b**, Maize seedling depicting the region harvested for manual explant preparation. **c**, Manually cut leaf tissue explants in *Agrobacterium* suspension. **d**,**e**, Leaf tissues in a permeable culture insert with excess *Agrobacterium* suspension being blotted off, and leaf tissues cultured on a filter paper sitting on surface of solid medium.

Encouraged by our results in maize and sorghum, we performed a preliminary evaluation of leaf transformation using nine species across the Poaceae, including *Eragrostis tef*, *Panicum virgatum* (switchgrass), *Cenchrus americanus* (syn. *Pennisetum glaucum*, pearl millet), *Setaria italica* (foxtail millet), *Triticum aestivum* (Pioneer Spring wheat), *Secale cereale* (rye), *Hordeum vulgare* (barley), *Saccharum officinarum* (sugarcane) and *Oryza sativa* (rice) (Fig. 4a). The method optimized for maize, but without the ancymidol or heat pretreatments of seedlings, was used for this assessment. Leaf tissue was harvested from the basal portion of the seedling and manually sliced into 1.5 to 3 mm fragments (Extended Data Fig. 2). The leaf pieces were then infected with *Agrobacterium* containing PHP97334 (Fig. 1a). Between six and 120 seedlings were used for different species/varieties (small numbers in some species were due to the limited availability of starting material). Using fluorescence microscopy to evaluate Zs*Green1* expression four to five days after infection, we observed good T-DNA delivery in all species tested (Fig. 4b, left micrographs). Further culture of the leaf tissue for four to five weeks resulted in fluorescent embryogenic calli (Fig. 4b, centre micrographs). After *Wus2*/*Bbm* excision, the tissue was cultured for three weeks on embryo maturation medium and then for two weeks on rooting medium, at which point regenerating plantlets were photographed (Fig. 4b, right photographs).

**Extended Data Fig. 3:**
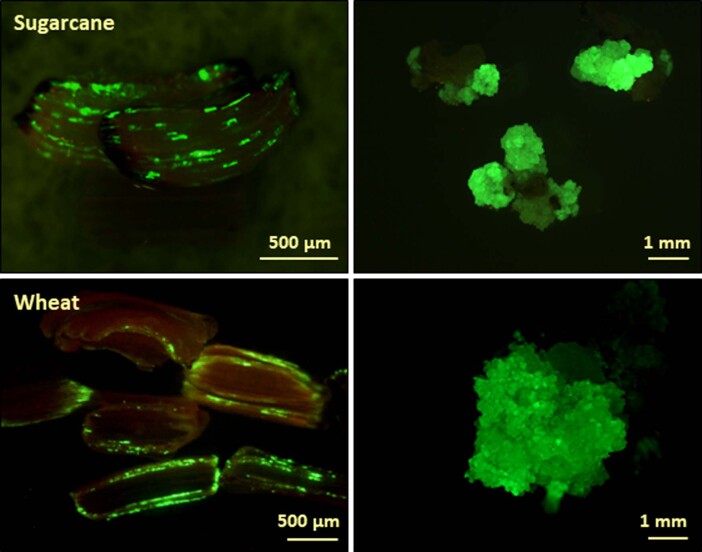
Transformation of *Saccharum officinarum* and *Triticum aestivum* leaf explants. Transient expression of ZsGreen1 three to four days after *Agrobacterium* infection (panels on left), demonstrating the relative T-DNA delivery. Green-fluorescent embryogenic callus formation three to four weeks after infection (panels on right). Images are representative of three independent experiments.

Embryogenic callus was produced in all species tested (the individual experiments for each species are summarized in Supplementary Table 8), and T_0_ plantlets with healthy shoots and roots were regenerated in seven of nine species (Fig. 4b). Two species, wheat and sugarcane, failed to produce T_0_ plants (Extended Data Fig. 3). In the case of Pioneer wheat variety PH456D, although somatic embryos were produced within the first 10–14 days (based on the early growth phenotype), embryogenic potential deteriorated and the callus became non-regenerable over the next 2–3 weeks. In the case of sugarcane, somatic embryogenic callus was produced and maintained, but embryogenic morphology was quickly lost after exposure to the 45 °C heat treatment used to stimulate Cre-mediated excision. The experiment with sugarcane will be repeated in the future, replacing the heat-inducible promoter with an abscisic acid/desiccation-inducible Rab17 promoter driving *Cre* to facilitate excision as described in Lowe et al.^10^.

## Discussion

The use of morphogenic genes to improve *Agrobacterium*-mediated transformation and/or Cas9-mediated gene editing in grass species continues to gain momentum. The combination of *Wus2* and *Bbm* to enhance immature embryo transformation in maize was first reported for several recalcitrant maize inbreds^10^. The application of these genes was further extended to sorghum transformation^10,12^, sorghum genome editing^37^, maize public inbred transformation^14,15,38^ and maize genome editing^15,35,36,39^. Recently, *Wox5* has also been demonstrated to improve transformation in numerous wheat varieties, with potential application in other cereals such as *Triticum monococcum*, triticale, rye, barley and maize^19^. Similarly, *Grf5* has been shown to improve transformation in maize inbred line A188^40^, and the *Grf4*/*Gif1* gene combination has been shown to stimulate growth and regeneration of transgenic events in wheat, rice and triticale^18^. However, the above progress continues to rely on immature embryo transformation, which has been a constraint for many grass family crops, motivating researchers to explore alternative explants for tissue culture and transformation.

The leaf base has been an attractive tissue culture explant for almost four decades, with callus initiation and regeneration being demonstrated in numerous Poaceae species including wheat^41–44^, oat^45,46^, barley^47,48^, rye^49^, maize^23^, rice^50^ and various apomictic grasses^51^. Accordingly, researchers have used leaf bases to generate embryogenic callus as the target explant for *Agrobacterium* infection and the regeneration of transgenic plants—for example, in maize^23^, indica rice^24,52^, Ma bamboo^25^ and teosinte^26^. However, except for a report in orchard grass (*Dactylis glomerata*)^53^ where transgenic plants were produced, studies where leaf-base tissue has been used as the direct transformation target were limited to demonstrating only transient transgene expression in rice^54^, wheat^55^ and maize^56^.

Lowe et al.^10^ have shown that the use of morphogenic genes *Wus2* and *Bbm* facilitated direct *Agrobacterium*-mediated transformation of basal leaf tissue and the recovery of maize plants with random transgene integration. Recently, this method has been extended to random transformation in *Panicum virgatum*^27^ and Cas9-mediated mutagenesis in *Eragrostis tef*^28^, demonstrating that *Wus2*/*Bbm* can facilitate direct leaf-base transformation in other grass species. However, to support the true potential of Cas9-mediated genome editing, substantially higher transformation rates are still required to produce the large numbers of plants necessary to recover complex genome modifications, such as targeted HDR-mediated gene insertion and large-scale chromosomal rearrangements^9^.

To address this need, we show that *Agrobacterium*-mediated delivery of *Wus2* and *Bbm* regulated by new promoters can significantly elevate leaf-base transformation in maize and sorghum. Previously, two promoter combinations have been used for *Wus2*/*Bbm*-mediated transformation of immature embryos. In the first one, the use of the *Nos* and *Ubi* promoters driving the expression of *Wus2* and *Bbm*, respectively, stimulated the development of embryogenic callus but required weeks of culture followed by morphogenic gene excision prior to plant regeneration^10,12^. The second promoter combination, *Axig1* for *Wus2* and *Pltp* for *Bbm*, stimulated rapid somatic embryo formation and did not require later excision^11,14,15,57^. Lowe et al.^10^ demonstrated that maize leaf transformation using *Nos*::*Wus2* and *Ubi*::*Bbm* initiates weak embryogenic callus that barely develops within the first two weeks after infection (Table 1, PHP81858), and the use of *Axig1*::*Wus2* and *Pltp*::*Bbm*, although leading to strong transient expression in leaf explants, did not result in subsequent somatic embryo formation (Table 1, PHP83652). This led us to test many different promoter combinations, some of which produced somatic embryos at slow (++) or faster (+++) rates (Table 1). Although these promoter combinations are not optimal for maize, they may prove useful in other grass species with further experimentation. The best results were obtained for a group of constructs when *Nos*, *Actin* or *Ubi* promoters controlled *Wus2* expression and *Bbm* was regulated by a *FMV*::*PCSV*::*MMV*::*Ubi* promoter (three viral enhancers upstream of the *Ubi* promoter). For maize and sorghum, we focused on *Nos*::*Wus2* and 3x*Enh–Ubi*::*Bbm* for further method development.

**Extended Data Fig. 4:**
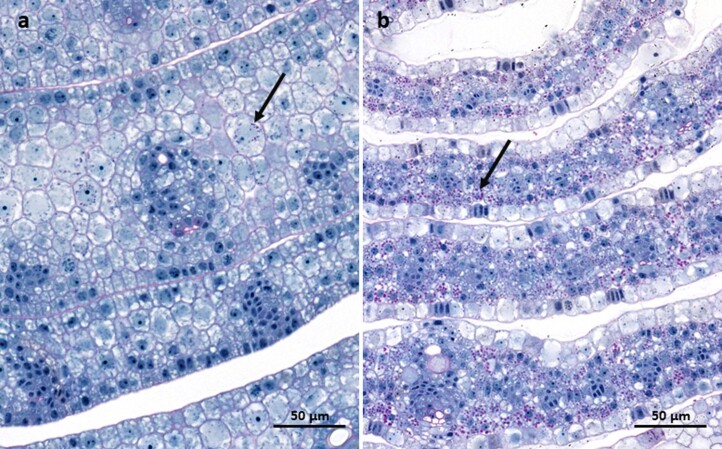
Sections of maize leaf base tissue from 14 day-old seedlings grown in the absence (a) or presence (b) of 2 mg/l ancymidol. **a**,**b**, Sections of maize leaf base tissue from 14 day-old seedlings grown in the absence (**a**) or presence (**b**) of 2 mg/l ancymidol. Sections are stained with periodic acid-Schiff for polysaccharides and aniline blue-black for protein. Arrow indicates magenta-stained amyloplasts. Note extensive presence of amyloplasts in developing mesophyll of ancymidol-treated leaf tissue and general lack of amyloplasts in tissue grown in the absence of ancymidol. Scale bar = 50 µm.

Transformation frequencies were further enhanced by growing seedlings on ancymidol and providing heat pretreatment before leaf explant harvesting and *Agrobacterium* infection. The benefit of heat exposure immediately before infection is similar to that seen for immature embryo transformation in rice^58^, maize^44,58^ and sorghum^32^, where it has been suggested that the accumulation of heat shock proteins may mitigate against the stress of *Agrobacterium* infection. A link between ancymidol exposure and stress mitigation is less clear. Instead, one possible explanation for the enhanced embryogenic response may be related to the observation that ancymidol-treated leaf bases accumulate noticeable amounts of starch in the developing mesophyll cells (Extended Data Fig. 4). A correlation between starch accumulation and improved somatic embryogenesis has been noted in other systems^59–61^, with the suggestion that starch is an easily metabolizable source of energy to support somatic embryo development. As a result, for PH1V69 we achieved average transformation frequencies (the number of T_0_ regenerants per starting seedling) of 260% with ancymidol and 560% with a combination of ancymidol and heat pretreatment (Table 2c). The protocol developed for PH1V69 was also successfully used to transform Pioneer NSS inbred PHR03 and public line B104 with frequencies around 200% and 180%, respectively; other tested NSS lines had transformation frequencies between 13 and 190%. Currently, we do not have a common metric to compare the efficiency of immature embryo transformation with that of seedling-derived leaf transformation simply because the explants used for *Agrobacterium* infection are so different. Also, when using the mini-Cuisinart for mechanized leaf fragmentation, the population of individual leaf pieces produced is too variable in number and size to provide a reproducible starting point to calculate efficiency. We have thus resorted to using the number of starting seedlings as our common denominator when comparing leaf transformation frequencies between experiments, between inbreds and between grass species. On the basis of this measure, we can unequivocally say that maize inbred PH1V69 and sorghum variety Tx430 produced high numbers of T_0_ transgenic events per starting seedling and that the other inbreds tested (such as B104) produced lower results. For B104 and other inbreds, leaf transformation frequencies will probably continue to improve as more laboratories work with this tissue for *Agrobacterium* infection and as new improvements (such as a modified ternary plasmid system for *Agrobacterium*-mediated immature embryo transformation developed in B104 (ref. ^62^) are brought to bear on leaf tissue.

Direct leaf transformation provides other advantages allowing automation and improved handling of the tissue culture material. Specifically, the tedious embryo isolation procedure is replaced with simple seedling-derived leaf tissue preparation, fragmentation is automated using a blender (potentially reducing variability in explant preparation) and *Agrobacterium* infection happens directly in the blending vessel. Moreover, while immature embryos are subcultured by transferring each embryo onto new medium individually, the transformed leaf fragments are placed on filter paper, which can be moved easily from one medium to the next, substantially reducing time and labour. Combined with eliminating the need for year-round embryo production in addition to seasonal variation that has plagued immature embryo methods for years^15^, these advantages make transformation easier, more practical, reproducible, ergonomically friendly and widely applicable to a broad variety of important grass species.

From the earliest days of immature embryo culture and transformation in grass species, it was always abundantly clear that somatic embryo formation occurred on the scutellar epithelium^63,64^. However, for leaf-base culture, the origin of the culture response remained unresolved. Despite many reports on leaf-base tissue responding to in vitro culture manipulations and callus production across a variety of Poaceae species^24,25,43,47,50^, the specific cell types involved were not clear. Our histological analysis along with observations of fluorescing multicellular clusters five to seven days after *Agrobacterium* infection (Extended Data Fig. 1) confirm earlier observations in Lowe et al.^10^ that somatic embryos predominantly appear to form from interior leaf cells, an observation that is consistent with embryo growth from leaf tissue in orchard grass^65^ and with observations in rice leaves constitutively expressing the Os*Bbm1* gene^66^. On the basis of the abundant early formation of fluorescent multicellular clusters (Extended Data Fig. 1a–c) and our current frequency of recovering transgenic plants from only a fraction of such early growth responses, there is probably a potential for further improvement of this method by simply reducing the rate of attrition during the culture process.

We also demonstrated that the leaf-base transformation level achieved in Pioneer proprietary maize SS inbred PH1V69 supports the recovery of plants with various Cas9-mediated genome modifications (NHEJ-based mutagenesis, gene dropouts and HDR-facilitated targeted gene insertions) at frequencies that approach ones obtained for immature embryos^35,36,39^. Another important aspect to consider for genome editing is the contribution of growth stimulation by morphogenic genes, concomitantly accelerating cell cycle progression and cell division, which contribute to the overall efficiency of Cas9-mediated modifications. Accordingly, the advantage of using *Wus2*/*Bbm* in immature embryos has been demonstrated in maize to facilitate the recovery of gene dropouts^35,34^ and HDR-mediated integration events^36,39^. Furthermore, the frequency of recovered gene dropouts in T_0_ sorghum plants is enhanced by *Wus2* beyond the level that can be accounted for by simply producing more T_0_ plants^17^. As speculated by Peterson et al.^36^, an added advantage of using morphogenic genes in genome modification experiments is the stimulation of cell division, providing a cellular environment conducive to more efficient complex forms of genome editing, including gene dropouts and HDR-mediated insertions. Our results with maize leaf bases are consistent with these interpretations.

In summary, our results clearly demonstrate the potential advantages of using leaf bases as the starting explant for transformation and genome editing in maize and sorghum. For maize, we have shown the potential of the method in terms of high efficiencies for both transgenic T_0_ plant production and genome editing. Moreover, successful leaf transformation and plantlet regeneration were demonstrated for multiple maize inbreds and a representative group of grass species. A single method can now be used across a wide spectrum of grasses, opening up the possibility of expanding transformation and genome editing methods across the entire Poaceae family.

## Methods

### Plant material

Seeds of Pioneer proprietary *Zea mays* lines PH1V69, PHR03, PH4257, PNSS01, 1PYWK36 and 1PEEA63 were used in the transformation experiments, in addition to the B104 public line. For *Sorghum bicolor*, seeds of line Tx430 were used. Transformation was carried out on additional Poaceae species: *Eragrostis tef* (teff variety DZ-01-354), *Panicum virgatum* (switchgrass variety Performer), *Pennisetum glaucum* (syn. *Cenchrus americanus*, pearl millet variety ICMB93333), *Setaria italica* (foxtail millet), *Triticum aestivum* (Pioneer Spring wheat variety PH456D), *Secale cereale* (rye, winter varieties Emerald and Pierre), *Hordeum vulgare* (barley, varieties Golden promise and Morex SPDL2), *Saccharum officinarum* (sugarcane variety CPCL02) and *Oryza sativa* (both indica variety IRV95 and japonica variety Kitaake).

### In vitro seed germination and preparation of leaf tissue explants

Mature seeds were surface sterilized in an 80% ethanol solution for three min, followed by incubation in 30% Clorox bleach solution containing 0.1% Tween-20 for 20 min and finally rinsing three times for five min each in autoclaved sterile water. The sterilized seeds were transferred onto solid 90O medium to germinate (Supplementary Table 9). In vitro germination and seedling growth were carried out under 80 μmol m^−2^ s^−1^ light intensity at 28 °C with a 16 hour light/eight hour dark photoperiod.

### Seedling pretreatments prior to harvesting leaf tissue for transformation

#### Ancymidol pretreatment

Surface-sterilized seeds were sown onto germination medium containing either no ancymidol (control medium 90O containing 0 mg l^−1^ ancymidol) or 90O medium plus 2 or 4 mg l^−1^ ancymidol (Sigma Aldrich, product number A9431). The germination and growth of seedlings occurred under 80 μmol m^−2^ s^−1^ light intensity using a 16-hour photoperiod at 28 °C, and the seedlings were harvested for transformation at 14 days for all replicate experiments and treatments.

#### Heat pretreatment

Surface-sterilized seeds were sown onto germination medium 90O plus 2 mg l^−1^ ancymidol. The germination and growth of seedlings occurred as described above. Before explant harvest, 12- to 18-day-old seedlings were placed into a 45 °C/70% RH incubator for three hours.

### Preparation of *Agrobacterium* suspension

In prior studies, the combination of helper plasmid pVIR9 (PHP71539) in *Agrobacterium* strain LBA4404 THY- has been highly efficacious for use in maize transformation^29^ and has been an integral component in optimizing *Wus2*/*Bbm* methods for immature embryos^10,11,16^ and now leaf tissue. *Agrobacterium tumefaciens* strain LBA4404 TD THY- harbouring helper plasmid PHP71539 and a binary donor vector was streaked out from a −80 °C frozen aliquot onto solid 12R medium (Supplementary Table 10a) and cultured at 28 °C in the dark for two days to make a master plate. A working plate was prepared by streaking four or five colonies from the 12R-grown master plate across fresh 810K medium (Supplementary Table 10b) and incubating overnight in the dark at 28 °C prior to using it for *Agrobacterium* infection. Immediately prior to seedling leaf tissue preparation, *Agrobacterium* was suspended and dispersed in 700F infection medium (Supplementary Table 11b).

If the seedlings were manually dissected (see below), a small volume of infection solution (700F) was prepared by mixing 10 ml of 700J medium (Supplementary Table 11a), 20 µl of acetosyringone (100 mM stock) and 0.02% (v/v) surfactant (BREAK-THRU S 233, Evonik Industries GmbH) in a 50 ml conical tube. *Agrobacterium* was collected from the working plate, transferred to the infection medium in the 50 ml tube and then vortexed until uniformly suspended. The optical density (OD) of the suspension was measured at 550 nm and adjusted to an OD of 0.6. The final *Agrobacterium* suspension was aliquoted into Corning six-well plates containing 0.4 µm permeable culture inserts (Falcon, Part Numbers 353046 and 353090, respectively) with each well containing about 8 ml of the suspension.

For mechanized leaf fragmentation in a Cuisinart Mini-Prep Food-Processor (see below), the infection mixture (700F) was prepared by combining 100 ml of 700J medium, 200 µl of acetosyringone and 0.02% (v/v) BREAK-THRU S 223. Approximately one half to a full working plate of *Agrobacterium* was transferred to the 100 ml mixture and adjusted to an OD of 0.6 at 550 nm.

### Seedling leaf preparation and *Agrobacterium* infection

The seedlings were grown as described above. The leaf-base segment (an approximately 2.5–3.0 cm section above the mesocotyl) was harvested from each 12- to 18-day-old in-vitro-germinated seedling with sterilized scissors, and the outer leaf was peeled away and discarded. The remaining leaf-base cylinders were bisected longitudinally into two lengthwise halves using a sterile no. 10 scalpel blade. The bisected leaf whorl halves were then either further dissected manually or processed mechanically in a blender.

#### Manual leaf explant preparation and *Agrobacterium* infection

The bisected leaf whorl segments were cross-sectioned into smaller leaf explants (~3 mm), which were transferred into a permeable culture insert sitting in a six-well culture plate containing 8 ml of premade *Agrobacterium* suspension (medium 700F, Supplementary Table 11b) and infected for 20 min at room temperature (Extended Data Fig. 2). After infection, the culture insert containing the *Agrobacterium*-infected leaf segments was removed from the six-well plate and placed on an autoclaved dry filter paper to wick up and remove any residual *Agrobacterium* solution. The infected leaf segments were then transferred onto a fresh filter paper (VWR 7.5 cm diameter) resting on 710N solid co-cultivation medium (Supplementary Table 12). Forceps were used to evenly disperse the leaf segments on the filter paper and to ensure that they had enough room to grow. The infected leaf tissues were incubated at 21 °C in the dark for two days.

#### Mechanized leaf chopping and *Agrobacterium* infection in the Cuisinart Mini-Prep Food-Processor

The bisected leaf whorls were directly dropped into the bowl of a Cuisinart Mini-Prep Food-Processor (MODEL DLC-1SSTG, pre-sterilized using 70% ethanol) containing 100 ml of premade *Agrobacterium* infection medium (700F). For each experimental replicate, the total number of leaf whorls (corresponding to the total number of seedlings used to produce each bisected leaf whorl) varied; the number listed in the results table is the number of starting seedlings in each replicate. The leaf segments were chopped at the low-speed setting with ten quick pulses. The chopped leaf tissues were left in the container with the *Agrobacterium* suspension for 20 min and gently agitated two or three times during this infection period. After infection, all chopped leaf tissues in the suspension were poured into an autoclaved stainless-steel mesh sieve (Target.com item no. 53142252) to drain out the *Agrobacterium* suspension. The infected leaf tissues were transferred onto two layers of autoclaved filter papers in an empty petri dish to blot-dry the residual *Agrobacterium* suspension. The infected leaf tissues from roughly one or two seedings were then transferred onto an autoclaved filter paper sitting on the surface of solid co-cultivation medium 710N and dispersed evenly. The infected leaf tissues were incubated at 21 °C in the dark for two days.

### Tissue culture, selection and regeneration

After two days of co-cultivation, the filter papers supporting the leaf segments were transferred from 710N to 13266P RM (Supplementary Table 13) and cultured for one week in the dark at 28 °C without selection. The filter papers subtending the tissue fragments were then transferred onto selection medium (13266P plus 150 mg l^−1^ G418 for *NptII* selection or 0.1 mg l^−1^ ethametsulfuron for *Hra* selection; see Supplementary Table 14a,b, respectively). After three weeks of culture on selection medium, the plates were placed into a controlled-temperature/humidity incubator (45 °C/70% RH) for two hours to stimulate the *Hsp17* promoter and induce Cre-mediated excision of *Wus2*, *Bbm* and *Cre* recombinase cassettes. The plates were removed from the incubator and kept at room temperature for one to two hours until the plates had cooled down.

After the heat treatment and temperature equilibration at room temperature, leaf segments with newly developed somatic embryos were transferred onto maturation medium 404 (Supplementary Table 15) without filter papers, cultured in the dark at 28 °C for two weeks and then moved into a 26 °C light room for an additional week. Leaf segments that now supported small shoots were transferred onto rooting medium 272M (Supplementary Table 16) for an additional two to three weeks until well-formed roots had developed, at which point the plantlets were ready for transfer to soil in the greenhouse. To ensure that each T_0_ plant was an independent event, only the most vigorously growing plantlet from each leaf piece (which often developed multiple somatic embryos) was transferred to rooting medium and finally the greenhouse.

### Molecular analysis of T_0_ plants

Molecular analyses of transformed leaf pieces for transcript levels of *Wus2* and *Bbm* were carried out as described previously^67^ with some modifications. Briefly, total RNA was isolated by grinding samples in RB Lysis Buffer (Omega Bio-tek), followed by adding an equal volume of 70% ethanol and transferring them to a Nunc Glass binding purification plate (Thermo Fisher Scientific) atop a 2 ml deep-well plate. The plates were covered with a Qiagen AirPore sheet (Qiagen), centrifuged and washed sequentially with RNA wash buffer I and II (Omega Bio-tek). RNA was eluted with nuclease-free water heated to 95 °C after a two min incubation in the Nunc Glass binding plate followed by centrifugation to collect the RNA in a 96-well plate. DNA was removed from the RNA samples using Roche Recombinant RNase-free DNAse I (Roche Diagnostics Corp.), and complementary DNA was synthesized using Applied Biosystems High Capacity cDNA Reverse Transcription kits (Thermo Fisher Scientific) according to the manufacturer’s instructions. Quantitative PCR was done using hydrolysis probes. The primers and probes were designed using Applied Biosystems Primer Express software. Hydrolysis-probe-based PCR was performed using Bioline Sensi-fast mix (Bioline) on an Applied Biosystem Viia7 Real-Time PCR instrument using the manufacturer’s conditions. Relative gene expression was calculated by normalizing against maize *Eukaryotic Initiation Factor 4-Gamma* gene (GenBank accession no. EU967723). A list of primers and probes used for the transcript analysis is given in Supplementary Table 17.

Molecular analyses of dropout and HDR-mediated gene insertion events were carried out as referenced in Peterson et al.^36^. Quantitative PCR was performed with QuantiTect Multiplex PCR Master Mix (Qiagen) according to the manufacturer’s directions (except for HR1/HR2 detection) on a ViiA 7 Real-Time PCR System (Thermo Fisher Scientific). Supplementary Table 18 lists the primers and probes used to detect *Wx1* dropout products, HR1/HR2 junctions, T-DNA components and HDR target site mutations. To detect *Wx1* dropout products (110 base pairs), endogenous *Wx1* target sites (3.8 kilobases) were flanked by primers/probes. The primers and probes were used to characterize T-DNA expression cassette components (*Wus2, BBM, Cas9*, gRNA and *Hra*) for T_1_ segregation copy number as well as HDR-mediated insertion product components (SiUbi promoter, *NptII* and SiUbi terminator). T_1_ qPCR characterization provides the best understanding of event integrity when coupled with long insert spanning PCR using LongAmp Hot Start Taq 2X Master Mix (New England Biolabs) followed by agarose gel electrophoresis. Genomic primers (forward primer 5′-GCGTGCGTGCTTACATGATG-3′ and reverse primer 5′-GTGCGACATTAAACAGTGTTAGTTGTAGCC-3′) that flank the HDR target site were designed to amplify both the intact insert product (~5.0 kilobases) and the allele without insertion (1.0 kilobases). Various insertions or deletions indicated by varied band sizes exist and may represent double-stranded break repair via the NHEJ pathway^36^. The detection of HR junctions was also completed with fluorogenic probe-based detection. Although the assays (Supplementary Table 18) spanned from the genomic region into the insertion products (HR1 (600 base pairs) and HR2 (586 base pairs)), modifications to the standard qPCR cycling protocol allowed for the digital detection of HR products. The following cycling parameters in a three-step cycling approach were used: 95 °C denaturation for 15 mins followed by 40 cycles of 95 °C/20 s denaturation, 62 °C/60 s annealing and 68 °C/30 s extension. The qPCR reaction mix contained 50 ng of template DNA, 900 nM primers and 100 nM probe. Although the HR1 product contained additional fluorescent background, the use of a proprietary normalizer (VIC) of similar size provided qPCR-based copy number prediction for both junctions. The background in the HR1 reaction was subtracted out on the basis of higher quantitation values. For T_1_ long-PCR-positive segregation events, the full insert products were Sanger sequenced with the primers noted in Supplementary Table 18. SbS analysis was performed as described by Zastrow-Hayes et al.^30^.

### Statistics and reproducibility

Comparison of means for all pairs was done by one-sided Tukey–Kramer honestly significant difference tests using JMP version 16.0.0 (SAS Institute Inc.). The numbers of replicates used in each experiment are either described in the legends of the respective figures and tables or presented as individual data points in the tables.

### Histology of leaf tissue

Non-transformed leaf tissue was collected after the tissue was cut into small fragments and fixed for histological examination (see below). Transformed leaf tissue pieces at five, ten and 15 days after infection were collected and placed into freshly prepared fixative, 2.5% glutaraldehyde (Electron Microscopy Sciences) in phosphate buffer, pH 7.0, and fixed overnight at room temperature. The tissue was washed in three changes of phosphate buffer (three to five min per change), and the tissue pieces were dehydrated in a graduated ethanol series to 100% ethanol. The material was infiltrated with activated Technovit 7100 (Heraeus Kulzer GmbH), starting at a 1:1 mixture of 100% ethanol:Technovit 7100 for one hour, followed by two changes of 100% Technovit 7100, the first for one hour and the second overnight. The samples were polymerized in polytetrafluoroethylene moulds after the addition of 1 ml Technovit 7100 hardener to 15 ml of activated Technovit 7100. Polymerization was allowed to proceed overnight under vacuum in a vacuum desiccator.

Sections were cut at 2.5 µm with a sapphire knife on a microtome (Leica 2050, Leica Biosystems). The sections were floated on drops of distilled water on glass slides (Fisherbrand SuperFrost Plus) and dried onto the slides on a hot plate at 50 °C. The sections were stained with periodic acid Schiff reagent for polysaccharides^68^ followed by a five min counterstain for proteins with 0.1% aniline blue black (in 7.0% acetic acid)^69^. Images were captured on a Nikon E800 microscope equipped with a Nikon DS-Ri1 camera (Nikon).

### Reporting summary

Further information on research design is available in the Nature Portfolio Reporting Summary linked to this article.

## Supplementary information

Supplementary Information Supplementary Tables 1–18, description of histology methods and references.

Reporting Summary

## Data Availability

The authors declare that all data supporting the findings of this study are available within the Article and its Supplementary Information files and Extended Data. Corteva Agriscience will provide plasmids to academic investigators for non-commercial research under an applicable material transfer agreement subject to proof of permission from any third-party owners of all or parts of the material and to governmental regulation considerations. Completion of a stewardship plan is also required. The Pioneer maize inbred lines PH1V69, PH4257, PNSS01, 1PEEA63 and 1PYWK36 described in this research are proprietary.
