# Supplementary material for: Leaf transformation for efficient random integration and targeted genome modification in maize and sorghum
Source: Nat Plants. 2023 Feb 9;9(2):255–70. doi: 10.1038/s41477-022-01338-0 (PMC9946824; doi:10.1038/s41477-022-01338-0)
Supplement: Supplementary file 1 — Supplementary Tables 1–18, description of histology methods and references. [file 41477_2022_1338_MOESM1_ESM.pdf]

nature plants

Article

https://doi.org/10.1038/s41477-022-01338-0

Leaf transformation for efficient random integration and targeted genome modification in maize and sorghum

In the format provided by the authors and unedited

Wang et al. Supplementary Tables, Figures and Methods

Supplementary Table 1. Genetic components used in T-DNA vectors for transformation and/or genome editing, with references

**Component type**	**Label**	**Description**	**References**
**Promoters**	Zm-Hsp17	Maize Heat shock promoter	An unpublished Corteva Agriscience sequence
	Ubi	The maize ubiquitin promoter, the 5' UTR and the first intron	Christensen et al., 1992
	Sb-Ubi	The sorghum ubiquitin promoter, the 5' UTR and the first intron	Sorghum Ubiquitin Promoter, Isolated from Genomic Sequence (Sorghum Bicolor line P898012).
	Si-Ubi	The *Setaria italica* ubiquitin promoter, the 5' UTR and the first intron	Promoter of the Ubiquitin 1 gene of Setaria italica, an unpublished Corteva Agriscience sequence.
	Sb-ALS	The sorghum ALS promoter	SB-ALS promoter and 5'UTR, DOE-JGI Sbi v3.1, SBChr04, bases 49239164-49240031. DOE-JGI Sbi v3.1 corresponds to Sorghum bicolor BTx623 assembly v3.0.1 and gene annotation v3.1 available from Phytozome (http://phytozome.jgi.doe.gov/). Chromosome 4 of Sbi v3.1 is registered as NCBI accessions NC_012873.2 and CM000763.3
	Zm-U6	Maize U6 promoter used in gRNA expression cassette	Maize U6 PolIII promoter from B73 chromosome 8
	Nos	The *Agrobacterium*-derived nopaline synthase promoter	An, 1986
	GOS2	Promoter from maize ortholog of a Gene from Oryza sativa (GOS) encoding a Eukaryotic translation initiation factor SUI1	Maize GOS2 [Protein translation factor SUI1 homolog (Protein GOS2)] promoter (PCO643716) isolated from B73 genomic DNA, an unpublished Corteva Agriscience sequence.

	Pltp	Maize phospholipid transferase promoter	Lowe et al., 2018.
	Actin	Rice Actin promoter	McElroy et al., 1990.
	8xDR5-35S	8 repeats of the synthetic DR5 auxin responsive element with the -45 minimal 35S promoter	Ulmasov et al., 1997.
	PEPC1	ZM-PEPC1 PRO, phosphoenolpyruvate carboxylase promoter from *Zea mays*, reference sequence GRMZM2G083841.	Reference sequence GRMZM2G083841
	RUBISCO SSU	Maize promoter for the Ribulose bisphosphate carboxylase (RUBISCO) Small Subunit protein.	PCO653887
	CAB	Maize chlorophyll A-B binding protein promoter	Sullivan et al., 1989.
	CSVMV	Cassava vein mosaic virus promoter	Verdaguer et al., 1998.
	SCBV	Sugarcane bacilliform virus promoter	Sequence ID No. 1 from US10,227,598
	SWEET11	Promoter for the maize ortholog to At3g48740.1 (AtSWEET11, sugar transporter)	Sequence ID No. 304 from WO2022072335
	DIURNAL10	*Zea mays* Diurnal10 promoter.	Sequence ID No. 305 from WO2022072335
	DIURNAL11	*Zea mays* Diurnal11 promoter. Chlorophyll a-b binding protein1 promoter.	Sequence ID No. 306 from WO2022072335
	DIURNAL12	*Zea mays* Diurnal12 promoter. Promoter from a Thiazole biosynthetic enzyme 1-1, chloroplast precursor gene.	Sequence ID No. 299 from WO2022072335
**3' UTR Sequences**	IN2-2	The maize IN2-2 terminator	Hershey and Stoner, 1991

	PINII	The potato proteinase inhibitor II (pinII) 3'sequence	An et al., 1989
	U6 TERM	Maize U6 terminator used in gRNA expression cassette	3' poly-T sequence from the maize chromosome 8 U6 gene, an unpublished Corteva Agriscience sequence.
	Os-T28 3'	The T28 3' regulatory sequence from *Oryza sativa*	Bhyri et al., 2014 US20140130205 A1
**Selection marker genes**	*NptII*	Maize codon optimized Neomycin Phosphotransferase II	See GenBank sequence (i.e. MN380783)
	Zm-Hra	The maize ALS double mutant gene conferring herbicide resistance	Green et al., 2009
	Zs-GREEN	A gene encoding the Zs-GREEN protein from *Zoanthus sp.*	Matz et al., 1999
**Maize morphogenic genes**	*Zm-Wus2*	The maize *Wuschel2* (*Wus2*) gene	Lowe et al., 2007 US7256322
	Zm-Bbm	The maize *Baby boom* gene (*Bbm*)	Gordon-Kamm et al., 2006 WO2005075655
**Recombinase**	*Cre*	A maize-optimized CRE recombinase gene (originally from the P1 bacteriophage), with an inserted potato LS1 intron	Odell et al., 1990
**Recombinase Target Site**	loxP	The recombinase target site for the CRE recombinase from *E coli*	Odell et al., 1990
**Nuclease gene**	*Cas9*	Maize codon-optimized *Streptococcus pyogenes Cas9* endonuclease	Svitashev et al., 2015
**T-DNA Borders**	RB & LB	The *Agrobacterium* C-58 nopaline strain right border (RB) and left border (LB) sequences from pTiC58	Wang et al., 1984 (RB)<br>Gielen et al., 1999 (LB)

	3xENH	Triple viral enhancer (FMV:PCSV:MMV) used to boost UBI promoter strength	GenBank Accession Number MN380787, see below for base coordinates of individual enhancers.
**Enhancers**	FMV	Enhancer from *Figwort Mosaic Virus*	GenBank Accession Number MN380787, expression vector pHP87598, bases 1,597-1,798
	PCSV	Enhancer from *Peanut Chlorotic Streak Virus*	GenBank Accession Number MN380787, expression vector pHP87598, bases 1,805-1,988
	MMV	Enhancer from *Mirabalis Mosaic Virus*	GenBank Accession Number MN380787, expression vector pHP87598, bases 1,995-2,181
**Homology arms**	HR1	Homology arm 419 base sequence homologous to a Chr1 genomic segment 5’ to the cut site.	An unpublished Corteva Agriscience sequence
	HR2	Homology arm 378 base sequence homologous to a Chr1 genomic segment 3’ to the cut site.	An unpublished Corteva Agriscience sequence

Supplementary Table 2. Relative transcript levels of *Wus2* and *Bbm* regulated by different promoters in leaf tissue of maize inbred PH1V69

Description	Plasmid	*Wus2*	*Bbm*
Nos:*Wus2*/Ubi:*BBM*	PHP97978	9.9[a] ± 2.4	141.6 [f] ± 43.1
Nos:*Wus2*/3XEnhUbi:*BBM*	PHP97334	27.4 [b,e] ± 4.5	243.4 [g] ± 29.6
Actin:*Wus2*/Ubi:*BBM*	PHP95385	35.5 [c,e] ± 14	158.4 [f] ± 51
Actin:*Wus2*/3XEnhUbi:*BBM*	PHP96277	133.1 [d] ± 40.2	283.8 [g,h] ± 78.3

Transcript levels are represented as the mean relative levels (± SD) of five replicates.

Comparison of means for all pairs was done by one-sided Tukey-Kramer HSD test using JMP Version 16.0.0 (SAS Institute Inc.). Numbers with different letters are significantly different from each other ($p<0.05$), and numbers with the same letter are not significantly different.

Exact p values are as given below:

Wus2

PHP97978 vs PHP97334 – $p<0.0001$

PHP95385 vs PHP96277 – $p=0.0007$

PHP97978 vs PHP95385 – $p=0.0076$

PHP97334 vs PHP96277 – $p=0.0004$

Bbm

PHP97978 vs PHP97334 – $p=0.0024$

PHP95385 vs PHP96277 – $p=0.0161$

PHP97978 vs PHP95385 – $p=0.5794$

PHP97334 vs PHP96277 – $p=0.3109$

Supplementary Table 3. T0 seed-set data in maize and sorghum

Genotype	Number of T0s	Number of T0 producing seeds	Seed production (%)	Pollination	Average seed set (Total seeds/ears)	Seeds set range
Maize PH1V69	36	28	77.7%	Selfed	172 (1890/11)	80-292
				Carry In	195 (3319/17)	13-378
Sorghum TX430	23	21	91.3%	Selfed	2057 (43191/21)	667-3255

Supplementary Table 4. Southern-by-Sequencing (SbS) data

Genotype	Number of T0s sampled for SbS	Number of T0s SbS-pass	SbS-pass %	Number of T0s SbS-fail	SbS-fail %	Failure details
Maize PH1V69	36	27	75.0%	9	25.0%	4-SNP
						2-Unlinked fragments
						1-Linked fragment
						2-Backbone present
Sorghum TX430	23	17	73.9%	6	26.1%	1- Unlinked fragments
						1-Secondary PHP present
						3- Mismatching borders
						1- Multiple copies or loci

Supplementary Table 5. Public inbred line B104 transformation data

Inbred	Experiment	Number of seedlings	Number of T0s	T0 Freq	Single copy	Single copy %	Single copy BB-free	QE%	UQE%	Number of escapes
B104	1	7	12	171%	8	67%	4	33%	57%	0
	2	7	13	186%	7	54%	5	46%	71%	1
	Total (Average)	14	25	**179%**	15	**60%**	9	**36%**	**64%**	1

T0 Freq = (number of T0 plants / number of starting seedlings) x 100
Single copy = number of T0 plants containing a single copy T-DNA insertion
Single copy % = (number of single-copy T0 plants / number of T0 plants analyzed) x 100
Single copy BB-free = number of T0 plants with a single copy T-DNA insertion, with no detectable expression plasmid backbone sequence
QE% = quality event frequency = (number of single-copy T-DNA events with no expression plasmid backbone / number of T0 analyzed) x 100
UQE% = usable quality event frequency = (number of QE plants / number of starting seedlings) x 100
Total frequencies are denoted in bold font

Supplementary Table 6. Preliminary leaf transformation data for four additional Pioneer Non-Stiff-Stalk inbreds

Inbred	Plasmid	Promoter for *Wus2*	Promoter for *Bbm*	Number of Seedlings	Transformation Response[1]	Number of T0 Plants	T0 Freq
PH4257	PHP97334	Nos	3xEnh-Ubi	24	++	0	0%
PNSS01	PHP97334	Nos	3xEnh-Ubi	24	++	3	13%
1PYWK36	PHP97334	Nos	3xEnh-Ubi	21	++++	10	48%
1PYWK36	PHP96277	Actin	3xEnh-Ubi	21	++++	20	95%
1PEEA63	PHP97334	Nos	3xEnh-Ubi	21	++++	8	38%
1PEEA63	PHP96277	Actin	3xEnh-Ubi	21	++++	40	190%

[1] Transformation response scored as in Table 1 (scores of ++ or ++++ correspond to 30-50% or 60-80% of the leaf pieces producing somatic embryos within the first 14 days after *Agrobacterium* infection).

T0 Freq = (number of T0 plants/number of starting seedlings) x 100

Supplementary Table 7. Segregation data for three targeted integration events in Pioneer inbred PH1V69

T0 Plant	T1 plants analyzed	HR1/HR2 positive T1 plants	T-DNA positive T1 plants	HR1/HR2 positive and T-DNA negative T1 plants
1	32	18	15	10
2	32	13	16	5
3	32	14	15	8

HR1/HR2 = the number of T1 plants with positive qPCR results across both the HR1 and HR2 integration junctions
T-DNA positive T1 plants = number of plants with positive qPCR signals for the transgenes within the T-DNA
HR1/HR2 positive and T-DNA negative T1s = tested plants containing the targeted HDR integration in which the T-DNA had segregated away

Supplementary Table 8. Leaf transformation data using *Agrobacterium* strain LBA4404 THY- (with PHP71539) to deliver the T-DNA from the maize-optimized construct PHP97334 into leaf cells of different grass species, with the *Agrobacterium* infection, culture resting period, selection, maturation and rooting all being performed using maize-optimized media

Cultivars	Variety	Number of Seedlings	Number of Zs-Green1+ Callus Events	Number of Regenerated T0 Plants
Eragrostis tef (tef)	DZ-01-354	29	18	6
Secale cereale (rye)	Emerald	54	41	14
Secale cereale (rye)	Pierre	81	50	7
Cenchrus americanus (pearl millet)	ICMB9333	40	14	3
Oryza sativa spp Indica (IRV94)	IR64	22	18	8
Oryza sativa spp Kitaake	Kitaake	120	54	42
Panicum virgatum (switchgrass)	Alamo	6	11	7
Triticum aestivum (wheat)	PH456D	64	4	0
Hordeum vulgare (barley)	Morex	24	14	5
Hordeum vulgare (barley)	Golden promise	20	3	3
Setaria italica (foxtail millet)	Foxtail millet	20	15	11
Saccharum officinarum (sugarcane)	CPCL02	10	12	0

Supplementary Table 9. Seed Germination Medium (90O)

**Seed germination medium, in 100 x 15 mm petri dishes**	
MS Basal Salt Mixture (Sigma M5524)	2.165 g/L
Sucrose	20 g/L
Adjust pH to 5.6 with NaOH	
Sigma Agar (Sigma A7921)	5 g/L
**Autoclave, cool to 55°C, then add the ingredients listed below**	
Ancymidol stock (2 mg/mL)[1]	1.7 mL/L
Benomyl stock (50 mg/mL)[2]	1 mL/L
Meropenem trihydrate (5 mg/mL)[3]	2 mL/L

[1]Ancymidol, Sigma, product A9431, 100mg size. Dissolve 2 mg/mL in DMSO. Store at 4°C for up to 90 days. Optional to filter sterilize using DMSO-safe syringe.

[2]Benomyl stock (50 mg/mL): dissolve 500 mg Benomyl in 10 mL acetone; store in 4°C, for 2-3 months. Benomyl available from Sigma as methyl 1-(butylcarbamoyl)-2-benzimidazolecarbamate, product 381586.

[3]Meropenem trihydrate (Carbosynth AM16380; 5 mg/mL): dissolve 50 mg in 10 mL autoclaved water, may need to sonicate to dissolve; filter sterilize with 0.22 µm syringe filter; store at 4°C, 2-3 mo.

Supplementary Table 10. Media for *Agrobacterium* preparation before leaf transformation.

**10-a) *Agrobacterium* Master Plate 12R (AB medium plus Thymidine) in 100 x 15 mm petri dishes**	
Glucose	5 g/L
Adjust pH to 6.8 with NaOH, make 900 mL before autoclave	
Bactoagar	15 g/L
**Autoclave, cool to 55°C, add the stocks listee below**	
AB Salts[1]	50 mL/L
AB Buffer[2]	50 mL/L
$FeSO_4$ $7H_2O$ stock (5x)[3]	2 mL/L
Thymidine (50 mg/mL)[4]	1 mL/L
Spectinomycin (50 mg/mL)[5]	1 mL/L
Gentamicin (50 mg/mL)[6]	1 mL/L

[1]AB Salts (20x): 20 g/L ammonium chloride, 6 g/L magnesium sulfate heptahydrate, 3 g/L potassium chloride, 0.228 g/L calcium chloride. Filter sterilize and store in 4°C.

[2]AB Buffer (20x): 60 g/L potassium phosphate dibasic, 20 g/L sodium phosphate monobasic. Filter sterilize and store in 4°C.

[3]$FeSO_4$ $7H_2O$ stock (5x): 1.25 mg/mL. Filter sterilize and store in 4°C.

[4]Thymidine stock (Sigma T1895; 50 mg/mL): dissolve 500 mg Thymidine in 10 mL autoclaved water; filter sterilize; store in 4°C, for 2-3 months.

[5]Spectinomycin stock (Sigma S4014; 50 mg/mL): dissolve 500 mg Spectinomycin in 10 mL autoclaved water; filter sterilize; store in –20°C, for 2-3 months.

[6]Gentamycin stock (Gold Biotechnologies G-400; 50 mg/mL): dissolve 500 mg Gentamycin in 10 mL autoclaved water; filter sterilize; store in –20°C, for 2-3 months.

**10-b) *Agrobacterium* Working Plate (Medium 810K = YEP medium plus thymidine) in 100 x 15 mm petri dish**	
Peptone	10 g/L
Yeast extract	5 g/L
NaCl	5 g/L
Adjust pH to 6.8 with NaOH	
Bactoagar	15 g/L
**Autoclave, cool to 55°C, add below stocks**	
Thymidine (25 mg/mL)[1]	2 mL/L
Spectinomycin (100 mg/mL)[2]	1 mL/L
Gentamicin (50 mg/mL)[3]	1 mL/L

Supplementary Table 11. ***Agrobacterium*****/leaf tissue infection medium**

a) Basal Medium (700J)

Magnesium sulfate, Anhydrous	1.204 g/L
Maltose	5 g/L
Adjust pH to 5.6 with NaOH	
**Autoclave, cool; can be stored in 4°C for 2-3 months**	

Use below to make infection medium.

b) Infection medium (700F)

Make fresh immediately before each day's transformation

700J basal liquid	100 mL
Thymidine (50 mg/mL)	100 µL/100 mL
Acetosyringone (100 mM)[1]	200 µL/100 mL
Break-thru (10% v/v)[2]	100 µL/100 mL

[1]Acetosyringone stock (Sigma D134406; 100 mM): dissolve 196.2 mg AS in 10 mL DMSO; no sterilization needed; store in –20°C, for 2-3 months.

[2]Break-Thru, Keep at room temperature and in a dark place.

Supplementary Table 12. Co-cultivation medium (710N)

**Co-Cultivation medium (710N) in 100 x 15 mm petri dishes**	
MS Basal Salt Mixture (Sigma M5524)	4.33 g/L
Myo-inositol	0.1 g/L
L-proline	0.7 g/L
Maltose	20 g/L
Glucose	10 g/L
Nicotinic acid (1 mg/mL)	0.5 mL/L
Pyridoxine (1 mg/mL)	0.5 mL/L
Thiamine (0.4 mg/mL)	2.5 mL/L
Cupric sulfate (100 mM)	0.049 mL/L
2,4-D (0.5 mg/mL)	4 mL/L
MES	0.5 g/L
**Adjust pH to 5.6 with NaOH, then add**	
Sigma Agar (Sigma A7921)	8 g/L
**Autoclave, cool to 55°C, then add the ingredients listed below**	
Acetosyringone (100 mM)	1 mL/L
Ascorbic acid (10 mg/mL)	1 mL/L
Silver nitrate (2 mg/mL)[1]	1.7 mL/L
Thymidine (25 mg/mL)	2 mL/L

[1]Silver nitrate stock (Sigma #S7276): Add 200 mg to 100 ml of deionized water. Store in dark in the fridge at 4°C for 90 days. Media containing silver nitrate should also be stored in the dark.

Supplementary Table 13. Resting medium (13266P)

Resting medium (13266P) in 100 x 15 mm petri dishes	
MS Basal Salt Mixture (Sigma M5524)	4.33 g/L
N6 Macronutrient Stock[1]	60 mL/L
B5H Minor Salts (1000x)[2]	0.6 mL/L
NaFe EDTA for B5H (100x)[3]	6 mL/L
Eriksson's Vitamins	0.4 mL/L
S&H Vitamins powder	0.6 g/L
Potassium Nitrate KNO_3	1.68 g/L
Thiamine (0.4 mg/mL)	0.5 mL/L
L-proline	1.98 g/L
Casein Hydrolysate (acid)	0.3 g/L
2,4-D (0.5 mg/mL)	1.6 mL/L
Sucrose	20 g/L
Glucose	0.6 g/L
**Adjust pH to 5.6 with NaOH, then add**	
TC Agar (Phytotech A296)	6 g/L
**Autoclave, cool to 55°C, then add the ingredients listed below**	
Dicamba (1 mg/mL)	1.2 mL/L
Silver Nitrate (2 mg/mL)	1 mL/L
Meropenem (5 mg/mL)[4]	10 mL/L

[1]N6 Macro Nutrient Stock: $CaCl_2 \cdot 2H_2O$, 1.66 g/L; $(NH_4)_2SO_4$, 4.62 g/L; KH_2PO_4, 4 g/L; $MgSO_4 \cdot 7H_2O$, 1.85 g/L; KNO_3, 28.3 g/L.

[2]B5H Minor Salts (1000x): Boric Acid, 3 g/L; $MnSO_4 \cdot H_2O$, 10 g/L; $Na_2MoO_4 \cdot 2H_2O$, 0.25 g/L; KI, 0.75 g/L.

[3]NaFe EDTA for B5H (100x): EDTA-$Na_2 \cdot 2H_2O$, 3.7 g/L; $FeSO_4 \cdot 7H_2O$, 2.79 g/L

[4]Meropenum purchased through Biosynth Carbosynth, brand Meropenem, product number AM16380.

Supplementary Table 14. Media for selection using a) G418, or b) ethametsulfuron, and medium for ABA-mediated induction of *Wus2*/*Bbm* excision in T-DNAs containing the Rab17:*Cre* expression cassette.

a) G418 selection using the *NptII* gene

13266P (autoclaved and cooled to approximately 45°C)	1 L
G418 (Phytotech G810)	150 mg/L
[1] G418 stock (Sigma A1720-5G 5mg): dissolve in water. Make 150mg/L stocks and store in -20°C.	

b) Ethametsulfuron selection using the *Hra* gene

13266P (autoclaved and cooled to approximately 45°C)	1 L
Ethametsulfuron (2 mg/mL)[1]	50 µL/L
[1]Ethametsulfuron stock (ChemService N-11866, 2 mg/mL): dissolve 10 mg in 1 mL DMSO aliquot and store at -20°C.	

c) ABA induction of the Rab17 Promoter driving *Cre* for excision of *Wus2/Bbm*

13266P (autoclaved and cooled)	1 L
Dicamba (1 mg/mL)	1.2 mL/L
Silver Nitrate (2 mg/mL)	1.7 mL/L
Meropenem (5 mg/mL)	2 mL/L
ABA (0.1 mM)	0.5 mL/L

Supplementary Table 15. Maturation medium (404)

Maturation medium (404) in 100 x 15 mm petri dishes	
MODIFIED MS BASAL SALTS	0.61g/L
Myo-inositol	0.1 g/L
L-proline	0.7 g/L
Sucrose	85 g/L
MS Vitamin Corteva Stock[1]	5 mL/L
Cupric sulfate (1 mg/mL)	1.25 mL/L
Ammonium Nitrate	1.2 g/L
Potassium Nitrate	2.52 g/L
Sodium Phosphate Monobasic	0.193 g/L
Zeatin (0.5 mg/ml)	0.06 ml/L
IBA (1mg/ml)	2 mL/L
**Adjust pH to 5.6 with NaOH, then add**	
TC Agar	6 g/L
**Autoclave, cool to 55°C, then add the ingredients below**	
MetaTopolin (0.5 mg/mL)	1 mL/L
Thidiazuron (1 mg/ml)	0.1 ml/L
ABA (0.1 mM)	1 mL/L
BAP (1 mg/mL)	1 mL/L
IAA (0.5 mg/mL)	2 mL/L
Meropenem (5 mg/mL)	2 mL/L

[1]MS Vitamin Corteva Stock: 0.4 g/L Glycine, 0.1 g/L Nicotinic acid, 0.1 g/L Pyridoxine HCl, 0.02 g/L Thiamine HCl.

Supplementary Table 16. Rooting medium.

**Rooting medium (272M)**	
MS Basal Salt Mixture (Sigma M5524)	4.33 g/L
Myo-inositol	0.1 g/L
Sucrose	40 g/L
MS Vitamin Corteva Stock[1]	5 mL/L
**Adjust pH to 5.6 with NaOH, than add**	
Bacto Agar (VWR BD Difco 214010)	6 g/L
**Autoclave, cool to 55°C, then add the ingredients below**	
IBA (1 mg/mL)	0.5 mL/L
Meropenem (5 mg/mL)	2 mL/L

[1]MS Vitamin Corteva Stock: 0.4 g/L Glycine, 0.1 g/L Nicotinic acid, 0.1 g/L Pyridoxine HCl, 0.02 g/L Thiamine HCl.

Supplementary Table 17. List of primers and probes used in transcript analysis of transformed maize leaf pieces

Gene	Forward primer (5' to 3')	Reverse primer (5' to 3')	MGB Probe (5' to 3')
Wus2	AGCAGATCCAGCGCATCAC	TGGAACCAGTAGAAGACGTTCTTG	CACGGCAAGATCGA
Bbm	AGATATGAGGCACATCTTTGGGATA	ACTTGACGACCCTTACGAGTTTG	AGTTGCAGAAGGGAAG
Eukaryotic Initiation Factor 4-Gamma (EIF4)	AACACTTAGGCCAGCATTCG	TGTCAAATGGCTCGAGGAG	CCGTTCTCCAAATCA

Supplementary Table 18. List of primers and probes used in the analysis of *Wx1* dropout and HDR-mediated gene insertion events

Assay	Forward primer (5' to 3')	Reverse primer (5' to 3')	qPCR probe (5' to 3')
Wx1 Dropout	GTGTGCGTGCGTGCAGAC	AGCAGGGATTATTTACTCCACCAC	6FAM-CAAGCCAAGGCGAGG MGB
Insertion HR1 junction	GCGTGCGTGCTTACATGATG	TAAGGTTAATAGATCCATCTCGCG	6FAM-CAGCTTAGCGGTTGTG MGB
Insertion HR2 junction	GTGCGACATTAAACAGTGTTAGTTGTAGCC	TGTGCTCTGCTCACATTGCG	6FAM-TCTCAAGGCTGTACCCAA MGB
NptII	CGTTGGCTACCCGTGATATTG	GGAAGCGGTCAGCCCATT	6FAM-TGAAGAGCTTGGCGGC MGB
Wus2	ATGCTCCACTGACGTTCCATAA	TGCCTCCTCCCGCTCC	6FAM-ACCGCCGCCCGCA MGB
Bbm	CGGCGATGTCTGCTTCAA	AAGCTCTGATCCCCTCATGCT	6FAM-ATCCCCCAAGATTG MGB
Cas9	CCAGCAGCTCCCCGAGA	GCCGTTTTTTGATTGGTCGA	6FAM-AGTACAAGGAGATCTTC MGB
gRNA	CTAATCACAAGAGTGGAGCGTACCTT	AGCCTTATTTTAACTTGCTATTTCTAGCTCT	6FAM-CCGAGCCGCAAGCA MGB
SB-UBITERM	ATGGTTGTTTTTGTTCGTCTCCTAATA	GGTCAAATGAACACCAGCCAAT	6FAM-TGCCTGGGATCAAAT MGB
SI-UBI1 PRO	CCCCACCGCCATAAATAGC	CAACACGAGACGAGATGAGATTG	6FAM-CCCTCGCCTTTCT MGB
SI-UBI TERM	GCTTGTGGTCGACTCCTGTTTC	AGTTAGACATTTGAGTTTGCCAGAAC	6FAM-ACTTGAGGCGTAACTC MGB
TS45 target site	CCACGGACTGGATTAGATAGTGGT	TCTAGCTTTGCATCATGTCTTGAAC	6FAM-ATTGCTCCTCATCCTCGA
Sanger sequencing primers			
F01	ATCATGCGTGTCGTTCGTACTC		
F02	ACGGAGGTAGTGCATCCTTTGT		
F03	CACTCCGGTGGTATATGTACTTAGG		
F04	AATTGTACGATGAAACTGTGCAGC		
F05	TTCCCCACCGCCATAAATAG		
F06	ACGGTTTACTGGATCATTGCCTAG		
F07	CCATGCTCTTGTTACTTGTTTTGGT		
F08	TGGTTGAACAAGATGGATTGCA		
F09	AGCACGTACTCGGATGGAAGC		
F10	TTGAAGTACCTGTGTCGGGATTG		
F11	GGATTTAAGCCTTCTCAGATTATGC		
F12	TGTCCACTCCAGAAGTCATTCCT		
F13	TTCAGAGCACATACACGCACATC		
R01	TGTTAGTTGTAGCCTTTGGCAATG		
R02	GATGTGCGTGTATGTGCTCTGAA		
R03	AGGAATGACTTCTGGAGTGGACA		

R04	GCATAATCTGAGAAGGCTTAAATCC		
R05	CAATCCCGACACAGGTACTTCAA		
R06	GCTTCCATCCGAGTACGTGCT		
R07	TGCAATCCATCTTGTTCAACCA		
R08	ACCAAAACAAGTAACAAGAGCATGG		
R09	CTAGGCAATGATCCAGTAAACCGT		
R10	CTATTTATGGCGGTGGGGAA		
R11	GCTGCACAGTTTCATCGTACAATT		
R12	CCTAAGTACATATACCACCGGAGTG		
R13	ACAAAGGATGCACTACCTCCGT		

Histology of leaf tissue

Non-transformed leaf tissue was harvested from seedlings and fixed for further histological preparation as described below. Transformed leaf tissue pieces at 5, 10, and 15 days after infection were collected and placed into freshly prepared fixative, 2.5% glutaraldehyde (Electron Microscopy Sciences, Hatfield, PA) in phosphate buffer, pH 7.0, and fixed overnight at room temperature. Tissue was washed in three changes of phosphate buffer (3-5 mins/change) and the tissue pieces were dehydrated in a graduated ethanol series to 100% ethanol. Material was infiltrated with activated Technovit 7100 (Heraeus Kulzer GmbH, Wehrheim, Germany), starting at a 1:1 mixture of 100% ethanol:Technovit 7100 for 1 h, followed by two changes of 100% Technovit 7100, the first for 1 h and the second overnight. Samples were polymerized in polytetrafluoroethylene (PTFE) molds after the addition of 1 ml Technovit 7100 hardener to 15 ml of activated Technovit 7100. Polymerization was allowed to proceed overnight under vacuum in a vacuum desiccator.

Sections were cut at 2.5 µm with a sapphire knife on a microtome (Leica 2050, Leica Biosystems, Deer Park, IL). Sections were floated on drops of distilled water on glass slides (Fisherbrand SuperFrost Plus) and dried onto the slides on a hot plate at 50°C. Sections were stained with periodic acid Schiff reagent for polysaccharides (O'Brien and McCully, 1981) followed by a 5 min counterstain for proteins with 0.1% aniline blue black (in 7.0% acetic acid) (Fisher, 1968). Images were captured on a Nikon E800 microscope equipped with a Nikon DS-Ri1 camera (Nikon, Melville, NY).

REFERENCES:

An, G. (1986). Development of plant promoter expression vectors and their use for analysis of differential activity of nopaline synthase promoter in transformed tobacco cells. Plant Physiol. 81:86-9.

An, G., Mitra, A., Choi, H.K., Costa, M.A., An, K., Thornburg, R.W., and Ryan, C.A. (1989). Functional analysis of transgene expression. Transgenic Res. 10:183-192.

Christensen, A.H., Sharrock, R.A., and Quail, P.H. (1992). Maize polyubiquitin genes: structure, thermal perturbation of expression and transcript splicing, and promoter activity following transfer to protoplasts by electroporation. Plant Mol. Biol. 18(4):675-689.

Fisher, D.B. (1968) Localization of endogenous RNA polymerase activity in frozen sections of plant tissues. J. Cell Biol. 39: 745-749.

Gielen J, Terryn, N, Villarroel R, Van Montagu M (1999). Complete nucleotide sequence of the T-DNA region of the plant tumor-inducing *Agrobacterium tumefaciens* Ti plasmid pTiC58. J. Exp. Bot. 50:1421-1422.

Green JM, Hale T, Pagano MA, Andreassi JL, II, Gutteridge SA (2009) Response of 98140 corn with gat4621 and hra transgenes to glyphosate and ALS-inhibiting herbicides. Weed Sci. 57:142–148.

Hershey HP, Stoner TD (1991) Isolation and characterization of cDNA clones for RNA species induced by substituted benzenesulfonamides in corn. Plant Mol Biol. 17(4):679-90.

Lowe K, La Rota M, Hoerster G, Hastings C, Wang N, Chamberlin M, Wu E, Jones T, Gordon-Kamm W (2018) Rapid genotype "independent" Zea mays L. (maize) transformation via direct somatic embryogenesis. In Vitro Cell Dev Biol Plant 54(3):240-252.

Matz M.V., Fradkov, A.F., Labas, Y.A., Savitsky, A.P., Zaraisky, A.G., Markelov, M.L., and Lukyanov, S.A. (1999) Fluorescent proteins from nonbioluminescent Anthozoa species. Nature Biotech. 17:969-973.

McElroy D, Zhang W, Cao J, Wu R (1990) Isolation of an efficient actin promoter for use in rice transformation. Plant Cell. 2(2):163-71.

O'Brien, T.P., and McCully, M.E. (1981) The Study of Plant Structure: Principles and Selected Methods. Termarcarphi Pty. Ltd., Melbourne, pp 357.

Odell, J., Caimi, P., Sauer, B., and Russell, S. (1990). Site-directed recombination in the genome of transgenic tobacco. Mol. Gen. Genet. 223:369-378.

Sullivan, T.D., Christensen, A.H. & Quail, P.H. (1998) Isolation and characterization of a maize chlorophyll a/b binding protein gene that produces high levels of mRNA in the dark. Molec. Gen. Genet. 215, 431–440.

Svitashev S, Young JK, Schwartz C, Gao H, Falco SC, Cigan AM (2015) Targeted Mutagenesis, Precise Gene Editing, and Site-Specific Gene Insertion in Maize Using Cas9 and Guide RNA. Plant Physiol. 169(2):931-45.

Ulmasov T, Murfett J, Hagen G, Guilfoyle TJ (1997) Aux/IAA proteins repress expression of reporter genes containing natural and highly active synthetic auxin response elements. The Plant Cell 9:1963-1971.

Verdaguer B, de Kochko A, Fux CI, Beachy RN, Fauquet C. Functional organization of the cassava vein mosaic virus (CsVMV) promoter. Plant Mol Biol. 1998 Aug;37(6):1055-67.

Wang K, Herrera-Estrella L., Van Montagu M, Zambryski P (1984) Right 25 by terminus sequence of the nopaline t-DNA is essential for and determines direction of DNA transfer from *Agrobacterium* to the plant genome. Cell 38:455-462.
