# Supplementary material for: Leaf transformation for efficient random integration and targeted genome modification in maize and sorghum
Source: Nat Plants. 2023 Feb 9;9(2):255–70. doi: 10.1038/s41477-022-01338-0 (PMC9946824; doi:10.1038/s41477-022-01338-0)
Supplement: Supplementary file 2 — Reporting Summary [file 41477_2022_1338_MOESM2_ESM.pdf]

Corresponding author(s): William Gordon-Kamm

Last updated by author(s): Nov 23, 2022

Reporting Summary

Nature Portfolio wishes to improve the reproducibility of the work that we publish. This form provides structure for consistency and transparency in reporting. For further information on Nature Portfolio policies, see our Editorial Policies and the Editorial Policy Checklist.

Statistics

For all statistical analyses, confirm that the following items are present in the figure legend, table legend, main text, or Methods section.

n/a	Confirmed	
☐	☒	The exact sample size (*n*) for each experimental group/condition, given as a discrete number and unit of measurement
☒	☐	A statement on whether measurements were taken from distinct samples or whether the same sample was measured repeatedly
☒	☐	The statistical test(s) used AND whether they are one- or two-sided<br>*Only common tests should be described solely by name; describe more complex techniques in the Methods section.*
☒	☐	A description of all covariates tested
☒	☐	A description of any assumptions or corrections, such as tests of normality and adjustment for multiple comparisons
☐	☒	A full description of the statistical parameters including central tendency (e.g. means) or other basic estimates (e.g. regression coefficient) AND variation (e.g. standard deviation) or associated estimates of uncertainty (e.g. confidence intervals)
☐	☒	For null hypothesis testing, the test statistic (e.g. *F*, *t*, *r*) with confidence intervals, effect sizes, degrees of freedom and *P* value noted<br>*Give P values as exact values whenever suitable.*
☒	☐	For Bayesian analysis, information on the choice of priors and Markov chain Monte Carlo settings
☒	☐	For hierarchical and complex designs, identification of the appropriate level for tests and full reporting of outcomes
☒	☐	Estimates of effect sizes (e.g. Cohen's *d*, Pearson's *r*), indicating how they were calculated

Our web collection on statistics for biologists contains articles on many of the points above.

Software and code

Policy information about availability of computer code

Data collection	Applied Biosystems Primer Express software v3.0.1 was used for primer and probe design
Data analysis	JMP software v16.0.0 (SAS Institute Inc.) was used for statistical analysis

For manuscripts utilizing custom algorithms or software that are central to the research but not yet described in published literature, software must be made available to editors and reviewers. We strongly encourage code deposition in a community repository (e.g. GitHub). See the Nature Portfolio guidelines for submitting code & software for further information.

Data

Policy information about availability of data

All manuscripts must include a data availability statement. This statement should provide the following information, where applicable:

- Accession codes, unique identifiers, or web links for publicly available datasets
- A description of any restrictions on data availability
- For clinical datasets or third party data, please ensure that the statement adheres to our policy

The authors declare that all data supporting the findings of this study are available within the article and its Supplementary Information files.

Human research participants

Policy information about studies involving human research participants and Sex and Gender in Research.

Reporting on sex and gender	N/A
Population characteristics	N/A
Recruitment	N/A
Ethics oversight	N/A

Note that full information on the approval of the study protocol must also be provided in the manuscript.

Field-specific reporting

Please select the one below that is the best fit for your research. If you are not sure, read the appropriate sections before making your selection.

☒ Life sciences ☐ Behavioural & social sciences ☐ Ecological, evolutionary & environmental sciences

For a reference copy of the document with all sections, see nature.com/documents/nr-reporting-summary-flat.pdf

Life sciences study design

All studies must disclose on these points even when the disclosure is negative.

Sample size	At least 10 individual seedlings were used for each replicate of maize and sorghum leaf transformation treatments, because based on our experience the downstream tissue culture work was manageable for one lab worker. Sampling size for numbers of T0 plants analyzed (72-370) for HDR experiments was established based on previous experience analyzing for CAS-mediated targeted integration events using maize immature embryos, as previously reported in Barone P, et al. Efficient Gene Targeting in Maize using Inducible CRISPR-Cas9 and Marker-Free Donor Template. Molecular Plant, (2020).
Data exclusions	No data were excluded from the analyses.
Replication	All experiments for maize and sorghum transformation were replicated at least 3 times. Screening of select Poaceae members for leaf transformation, given in Supplementary data, was carried out one time with number of seedlings used ranging from 6-120.
Randomization	For each experimental replicate, the total number of seedlings used were pooled for Agrobacterium infection and chopped in a food processor. The tissue fragments were then randomly distributed for further culture and selection. This provided internal randomization of samples for each experimental replicate.
Blinding	For each experiment, treatments were compared to a control treatment with no preconceived knowledge of whether the experimental variables being altered would have a positive or negative impact on the results. Beyond this, no additional blinding was used.

Reporting for specific materials, systems and methods

We require information from authors about some types of materials, experimental systems and methods used in many studies. Here, indicate whether each material, system or method listed is relevant to your study. If you are not sure if a list item applies to your research, read the appropriate section before selecting a response.

Materials & experimental systems

n/a	Involved in the study
☒	☐ Antibodies
☒	☐ Eukaryotic cell lines
☒	☐ Palaeontology and archaeology
☒	☐ Animals and other organisms
☒	☐ Clinical data
☒	☐ Dual use research of concern

Methods

n/a	Involved in the study
☒	☐ ChIP-seq
☒	☐ Flow cytometry
☒	☐ MRI-based neuroimaging
